# A temporal differentiation program after birth delineates γδ T cell immunity

**DOI:** 10.1126/sciadv.aee6790

**Published:** 2026-08-05

**Authors:** Maria V. Baglioni, Marcelo G. F. Fares da Silva, Andrea Sonnenholzner, John Rizk, Alastair Copland, Elisa Catafal-Tardos, Davide Secci, Paula Peñalver-Sebastián, Martin F. Laursen, Susanne S. Kappel, Jacob Oehlenschlæger, Mark B. Ellebæk, Gitte Zachariassen, Lise Aunsholt, David Bending, Lars R. Olsen, Vasileios Bekiaris

**Affiliations:** ^1^ Department of Health Technology, Technical University of Denmark, Kgs Lyngby, Denmark.; ^2^ Department of Immunology and Microbiology, LEO Foundation Skin Immunology Research Center, University of Copenhagen, Copenhagen, Denmark.; ^3^ Department of Immunology and Immunotherapy, College of Medicine and Health, University of Birmingham, Birmingham, UK.; ^4^ National Food Institute, Technical University of Denmark, Kgs Lyngby, Denmark.; ^5^ Department of Neonatal and Pediatric Intensive Care, Copenhagen University Hospital, Rigshospitalet, Copenhagen, Denmark.; ^6^ Department of Pediatric Surgery, Copenhagen University Hospital, Rigshospitalet, Copenhagen, Denmark.; ^7^ Research Unit for Surgery, Odense University Hospital and Department of Clinical Research, University of Southern Denmark, Odense, Denmark.; ^8^ Centre of Excellence in Gastrointestinal Diseases and Malformations in Infancy and Childhood (GAIN), Odense University Hospital, 5000 Odense, Denmark.; ^9^ Hans Christian Andersen Children’s Hospital, Odense University Hospital and Department of Clinical Research, University of Southern Denmark, Odense, Denmark.; ^10^ Department of Clinical Medicine, University of Copenhagen, Copenhagen, Denmark.

## Abstract

Type 1 and type 3 γδ T cells develop in the embryonic thymus, yet it is not known whether they continue to differentiate after birth in tissues. Here, we show that in the first week of life, intestinal γδ T cells up-regulated many T cell activation and type 3 genes, while the postweaning period was dominated by induction of genes related to effector function, type 1 immunity, and cytotoxicity. During this period, γδ T cells protected the gut from fungal infection. In the adult intestine, type 3 interleukin-17–producing γδ T cells (γδT17 cells) had a distinct phenotype, a Tbet-dependent type 1 immune profile and enhanced T cell receptor (TCR) signaling. Last, we found a γδT17 cell–like population in the neonatal human gut. Collectively, our data suggest that γδ T cells continue to mature after they leave the thymus and, upon localization to the gut, a dynamic temporal differentiation program reshapes type 3 cells into Tbet^+^ type 1 effectors with heightened TCR activity.

## Teaser

Intestinal γδ T cells undergo temporal immunological maturation during neonatal, postnatal, and adult life.

## INTRODUCTION

In the mouse, there are at least two functionally distinct innate γδ T cell subsets: a type 1 that produces interferon-γ (IFN-γ) and expresses the lineage transcription factor Tbet (γδT1) and a type 3 that produces interleukin-17 (IL-17) and expresses the lineage transcription factor retinoic acid receptor-related orphan receptor gamma t (RORγt) (γδT17) (*1*, *2*). Type 1 and type 3 bifurcation occurs in the embryonic thymus and is coordinated by the strength of the T cell receptor (TCR) signal, such that weak TCR signaling promotes type 3 programming, while strong signaling promotes type 1 programming (*3*–*6*). A number of animal studies have implicated γδT17 cells as pathogenic in inflammatory disease models (*7*–*9*) and cancer (*10*), which has drawn particular attention to this subset. These cells additionally undertake tissue-specific homeostatic roles, such as thermoregulation in adipose tissue (*11*) or the regulation of memory and anxiety in the brain (*12*, *13*).

The vast majority of murine γδT17 cells expresses either the Vγ6 or Vγ4 TCR chains [nomenclature according to Heilig and Tonegawa (*14*)] and can be found in most mucosal and barrier tissues as well as secondary lymphoid organs (*15*, *16*). In healthy humans, IL-17–producing γδ T cells are scarce in the blood (*17*, *18*) and tissues (*19*, *20*); however, they have been reported in inflammatory and infectious diseases such as psoriasis (*8*, *21*), multiple sclerosis (*22*), spondyloarthritis (*23*), candidiasis (*24*), and cancer (*20*). Recent single-cell RNA sequencing (scRNA-seq) data have provided evidence for the presence of precommitted type 3 Vγ9Vδ2 γδ T cells in the human embryonic thymus (*17*, *25*). In addition to Vδ2, both Vδ1^+^ and Vδ3^+^ cells have been shown to be able to produce IL-17 (*24*, *26*).

Despite the undisputed importance of neonatal life in imprinting life-long immunity (*27*), data linking γδ T cell functional and molecular maturation with temporal neonatal events are scarce. We know, for example, that the transcription factor signal transducer and activator of transcription 5 (STAT5) and the noncanonical nuclear factor κB pathway sustain the survival, proliferation, and function of neonatal γδT17 populations, and this affects subsequent adult immunity (*28*, *29*). Beyond γδT17 cells, the neonatal gut supports the maturation of Vγ7^+^ intraepithelial cells (*30*), while an interaction between γδ T cells and the microbiota is important to prevent type 2–driven inflammation in the lung and skin (*31*, *32*). The neonatal period appears to also support human γδ T cells with type 3 potential, which can be expanded from cord but not adult blood (*33*, *34*), while other human subsets rapidly expand and acquire effector function after birth (*35*, *36*). These data indicate a key role for early life signals in directing γδ T cell development and functional maturation. However, what are these signals, when is their temporal inclusion, and how they imprint tissue specific properties are not yet clear.

In the present study, we show that γδ T cells localize to the embryonic and neonatal gut, and we provide evidence that intestinal Vγ6 (γδT17 only) and Vγ4 (γδT17 and γδT1) cells undergo temporal transcriptional changes during the neonatal and postneonatal period characterized by vigorous immunological maturation during the first week of life and after weaning. Moreover, Vγ4 cells up-regulate expression of type 3 signature genes during the first week of life and type 1 and cytotoxicity effector genes after weaning. During this neonatal maturation period, intestinal γδ T cells could protect against fungal infection. In the adult intestine, γδT17 cells diversify from their lymph node (LN) counterparts by acquiring a type 1 transcriptional profile and the distinct phenotype Tbet^+^ICOS^+^P2RX7^+^ITGA1^+^SLAMF7^+^. Tbet is responsible for a major part of this type 1 programming and additionally regulates production of IFN-γ and IL-17A/F in response to IL-23 and IL-12. Tbet could be induced in newborn thymic γδT17 cells by a combination of TCR and cytokine signaling. We also found that unlike in the LN, γδT17 cells in the gut have an active TCR leading to productive signaling upon engagement. Last, we provide evidence that the human neonatal intestine contains a type 3 γδ T cell population resembling mouse intestinal γδT17 cells.

## RESULTS

### Neonatal but not embryonic intestinal γδT17 cells express Tbet and protect from infection

**Fig. 1.**
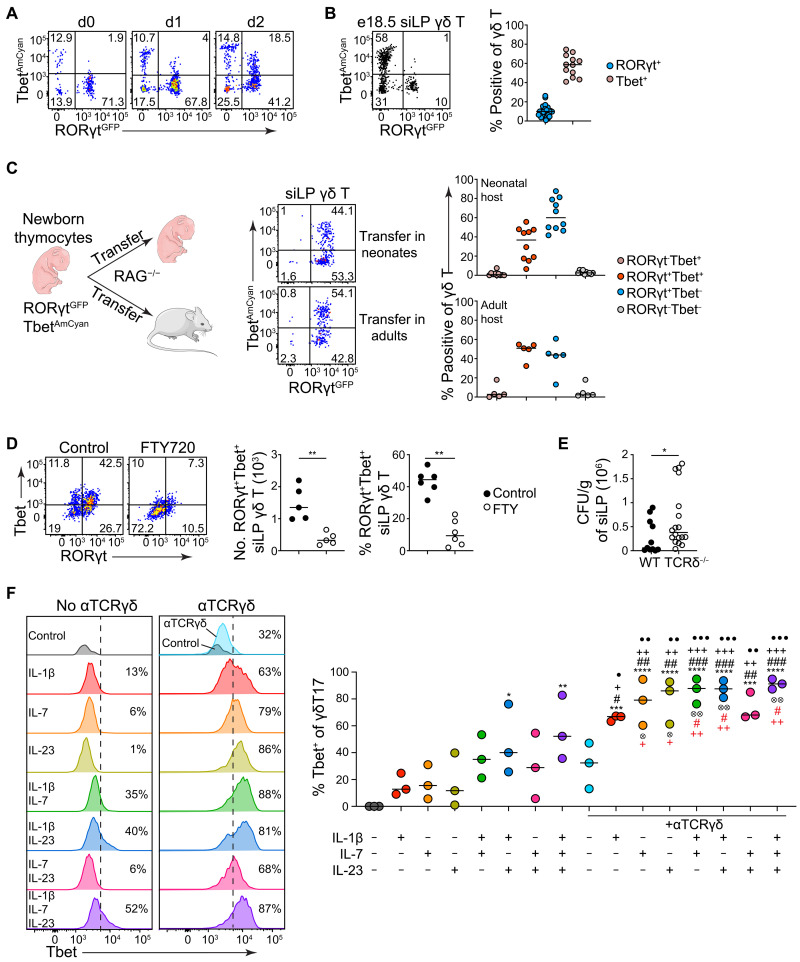
Neonatal but not embryonic intestinal γδT17 cells express Tbet and protect from infection. (**A**) Representative plots showing RORγt and Tbet expression in the siLP of RORγt^GFP^Tbet^AmCyan^ neonatal mice at the indicated day (d) after birth (two experiments, with six mice). (**B**) Representative plots and quantification of RORγt^+^ and Tbet^+^ γδ T cells from the siLP of E18.5 RORγt^GFP^Tbet^AmCyan^ embryos (three experiments, with 12 or 8 mice). (**C**) Schema of the experimental design and representative plots with corresponding quantification of the indicated siLP γδ T cell populations following transfer into neonatal or adult RAG1^−/−^ hosts (two neonatal and three adult experiments, with 5 or 10 mice). (**D**) Representative plots of RORγt and Tbet expression in siLP γδ T cells and quantification of numbers and frequencies of RORγt^+^Tbet^+^ cells after treatment with FTY720 (three experiments, with five to six mice). (**E**) Quantification of fungal burden in the siLP, expressed as colony-forming units (CFU) per gram of tissue, in mice orally infected with *C. albicans* and analyzed 4 days postinfection (two experiments, with 11 or 18 mice). (**F**) Representative histograms of Tbet expression in γδT17 cells from newborn thymus after 2-day stimulation under the indicated conditions and quantification (three experiments, each consisting of a pool of 9 or 12 newborn mice); numbers in histograms indicate frequency of positive cells from one representative experiment. In all graphs, each symbol represents a mouse and line the median. In (D) and (E) *P < 0.05; **P < 0.01 using the Mann-Whitney test. In (F), each symbol denotes the following statistical comparisons: * versus control; # versus *IL-1*β; + versus *IL-7*; • versus *IL-23*; ⊗ versus *GL3*; # versus *IL-1*β/*IL-7*; + versus *IL-7/IL-23* (*,#,+,•,⊗, #,+*P* < 0.05; **,##,++,••,⊗⊗, ++*P* < 0.01; ***,###,+++,•••*P* < 0.001; ****P < 0.0001), calculated using one-way analysis of variance (ANOVA) with Tukey’s multiple-comparisons correction.

We previously demonstrated that intestinal lamina propria (LP) γδT17 cells up-regulate Tbet during the first week of life (*28*). This raised the hypothesis that γδT17 cells may localize in the gut before birth and mature in situ thereafter. To test this, we analyzed embryonic and neonatal tissue from mice that transgenically expressed green fluorescent protein (GFP) and AmCyan under the control of the RORγt or Tbet promoters, respectively (RORγt^GFP^Tbet^AmCyan^). We used these mice so that we can trace both type 1 (Tbet^+^) and type 3 (RORγt^+^) populations, as well as the expression of Tbet in gut γδT17 cells (Fig. 1A). We found that ∼10% of all embryonic day 18.5 (E18.5) small intestinal LP (siLP) γδ T cells expressed RORγt, while, on average, 60% expressed Tbet (Fig. 1B). Thus, γδT17 cells seed the intestine already during embryonic life. To test whether the gut could also be seeded by γδT17 cells after birth, we isolated newborn thymocytes from RORγt^GFP^Tbet^AmCyan^ mice that were depleted of CD4^+^, CD8^+^, and TCRβ^+^ cells and transferred them into either adult or 2-day-old recombination activating gene 1-deficient (RAG1^−/−^) mice (Fig. 1C). After transfer, we analyzed siLP populations and could identify intestinal RORγt^+^ γδ T cells that had up-regulated Tbet (Fig. 1C). In these experiments, however, we could recover very few Tbet^+^RORγt^−^ cells, perhaps reflecting impaired capacity to migrate to the gut after transfer. In addition, treatment of newborn mice with fingolimod (FTY720), which blocks egress from the thymus, reduced the numbers and frequencies of RORγt^+^Tbet^+^ γδ T cells (Fig. 1D), suggesting contribution of the neonatal thymus toward the numbers of intestinal γδT17 cells. As γδ T cells have been linked before with protective responses against fungal infections (*37*), we tested whether they could protect the neonatal intestine from *Candida albicans*. Oral infection of neonatal wild-type (WT) and TCRδ^−/−^ mice showed that γδ T cells were required for optimal protection against *C. albicans* (Fig. 1E). Therefore, our data suggest that γδT17 cells home to the intestine during embryonic and neonatal life, after which they up-regulate Tbet and, together with other γδ T cell subsets, could play a protective role against early life fungal infections. What exactly triggers induction of Tbet after birth remains unknown. Using germ-free (GF) mice and mice sourced from a local pet store, we show that induction of Tbet was independent of the microbiota (fig. S1, A to D). However, Tbet could be induced in newborn thymic γδT17 cells when TCR activation was combined with the cytokines IL-1β, IL-7, and IL-23 (Fig. 1F). It is plausible that the cytokine milieu of the neonatal gut, together with putative TCR ligands, supports Tbet expression.

### Immunological maturation of intestinal γδ T cells in early life

**Fig. 2.**
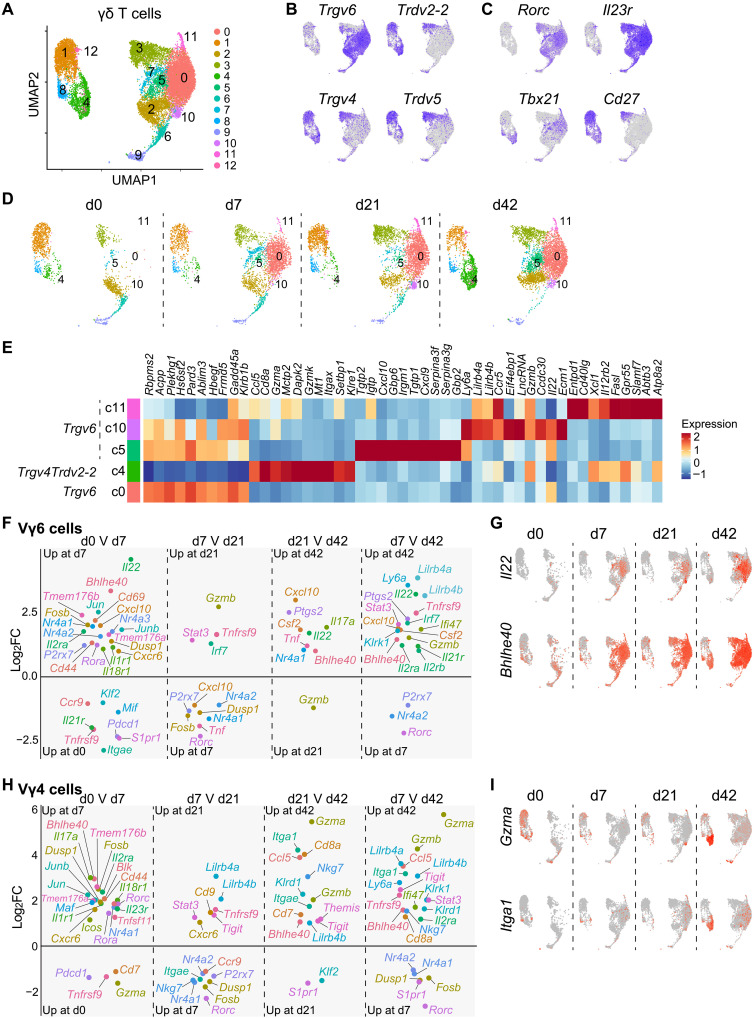
Immunological maturation of intestinal γδ T cells in early life. (**A**) γδ T cells were sorted from day 0 (10 mice; 1832 cells), day 7 (9 mice; 3294 cells), day 21 (3 mice; 4260 cells), and day 42 (2 mice; 5915) siLP, and the UMAP represents clustering of the cells after integration of all time points. (**B** and **C**) Feature plots showing expression of *Trgv* and *Trdv* chains (B) and representative type 3– and type 1–associated genes (C). (**D**) UMAP representation of γδ T cells across all time points, highlighting clusters c0, c4, c5, c10, and c11. (**E**) Heatmap showing expression of the top 10 genes for each cluster shown in (D). (**F** and **H**) Plots indicating selected DEGs and their log_2_ fold change (log_2_FC) between the indicated time points of Vγ6 (F) and Vγ4 (H) γδ T cells. (**G** and **I**) Feature plots showing expression of *Il22* and *Bhlhe40* (G) and *Gzma* and *Itga1* (I) across all time points. All shown genes were significantly expressed (*P* < 0.05) with a cutoff expression of log_2_FC of ≥1. *P* values were calculated using the Wilcoxon rank sum test. d, day.

The induction of Tbet shortly after birth suggested that there is differentiation of γδT17 cells in the intestine during neonatal life. To gain mechanistic insight regarding the molecular changes that occur throughout neonatal life, we isolated γδ T cells from the siLP of newborn (day 0), 7-, 21-, and 42-day-old mice and performed scRNA-seq. We used Uniform Manifold Approximation and Projection (UMAP) analysis to visualize γδ T cells by integrating all time points. Unsupervised clustering identified 13 clusters of which 7 (c0, c2, c5, c6, c7, c10, c11) were *Trgv6* (Vγ6^+^) and 4 (c1, c3, c4, c8) were *Trgv4* (Vγ4^+^), while a proliferating cluster (c9) was a mix of Vγ6^+^ and Vγ4^+^ cells (Fig. 2, A and B, and table S1). Of the Vγ4^+^ clusters, one was only *Trdv2-2*^+^ (c4), while the rest expressed a mix of *Trdv2-2* and *Trdv5* chains (Fig. 2B). All Vγ6^+^ clusters were type 3 as shown by expression of *Rorc* and *Il23r*, while Vγ4^+^ clusters were split into type 1 (shown by *Tbx21* and *Cd27* expression) and type 3 cells (Fig. 2C), as is the case in other organs (*38*). Temporal analysis showed that some clusters were absent or nearly absent at day 0 but appeared or expanded after birth (Fig. 2D). In particular, the Vγ6^+^ clusters c0, c5, c10, and c11 were only evident from day 7 onward, and the Vγ4^+^ cluster c4 expanded after weaning by day 42 (Fig. 2D). Of the top 10 genes that defined these clusters, many were associated with T cell activation and effector function, such as cytokines (e.g., *Il22*), chemokines (e.g., *Ccl5*), interferon-inducible genes (ISGs) (e.g., *Gbp6*), and cytotoxicity-related molecules (e.g., *Gzma*) (Fig. 2E and table S1).

The above suggested active maturation of the γδ T cell compartment during neonatal life and the postweaning period and prompted us to investigate in more detail temporal gene expression changes in Vγ6- and Vγ4-expressing cells. Therefore, we compared differentially expressed genes (DEGs) in a time-wise manner: days 0 to 7, days 7 to 21, and days 21 to 42. Within the first week of life Vγ6 cells up-regulated many genes related to immune function such as cytokines and cytokine receptors (e.g., *Il22*, *Il2ra*, *Il1r1*, and *Il18r1*), cell activation and tissue residency molecules (e.g., *Cd69* and *Cd44*), chemokines and chemokine receptors (e.g., *Cxcl10* and *Cxcr6*), signaling molecules including many associated with TCR activation (e.g., *Nr4a1*, *Nr4a2*, *Nr4a3*, *Dusp1*, and *P2rx7*), the RORγt target genes *Tmem176a* and *Tmem176b* (*39*), and transcription factors such as *Rora*, *Bhlhe40*, *Jun*, and *Junb* (Fig. 2F, fig. S2A, and table S2). Notable immune-related DEGs at day 0 included *Pdcd1*, the gut homing molecules *Itgae* and *Ccr9*, the tissue egress molecules *S1pr1* and *Klf2*, and the cytokine *Mif*, *Il21r*, and *Tnfrsf9* (4-1BB) (Fig. 2F, fig. S2A, and table S2). To substantiate these findings, we additionally sorted intestinal RORγt^+^ γδ T cells from 0- and 7-day-old RORγt^GFP^ mice and performed bulk RNA-seq. We found that at day 7, the cells had up-regulated many genes indicative of immunological maturation including the transcription factors *Tbx21*, *Bhlhe40*, and *Nr4a1*, the adhesion/migration molecules *Cxcr3*, *Ccl20*, *Cd44*, and *Cd69*, the cytokines *Tnf* and *Csf2* (granulocyte-macrophage colony-stimulating factor), and the signaling molecules *Tnfaip3* and *Map3k14* (fig. S2E and table S3), in agreement with our scRNA-seq data.

Between days 7 and 21, there were much fewer new induced genes, while day 7 genes retained their higher expression (Fig. 2F, fig. S2A, and table S2). This was also the case in the transition between days 21 and 42, although we noted induction of the cytokines *Il17a*, *Il22*, and *Csf2*, as well as *Ptgs2* (cyclooxygenase-2, COX-2) (Fig. 2F, fig. S2A, and table S2). These data suggested that the bulk of transcriptional maturation occurs during the first week of life, while later stages are more focused on sustaining and inducing genes related to effector function. Comparison of day 7 and day 42 cells showed that the latter had higher expression levels of a number of genes associated with effector function, ISGs, and the inhibitory receptors *Lilrb4a* and *Lilrb4b* (Fig. 2F, fig. S2C, and table S2). The high expression of at least some of these genes correlated with the clusters that appeared and expanded from day 7 onward, further demonstrating the presence of temporal maturation events (Fig. 2G).

When we interrogated Vγ4 cells, we found that their transition from birth to adulthood was similar to Vγ6 cells. Hence, by day 7, they had up-regulated the same genes or groups of genes, while from days 7 to 21, induction of new immune-related genes was reduced (Fig. 2H, fig. S2B, and table S2). An important difference between the two populations was the clear induction of type 3 genes at day 7 (e.g., *Rorc*, *Il17a*, *Maf*, *Il23r*, and *Blk*) in Vγ4 cells (Fig. 2H, fig. S2B, and table S2), indicating that part of their type 3 programming occurs during the first week of life. After weaning at day 42, Vγ4 cells up-regulated many type 1 and cytotoxicity-related genes (e.g., *Nkg7*, *Klrd1*, *Themis*, *Itga1*, *Gzma*, and *Cd8a*) (Fig. 2H; fig. S2, B and D; and table S2). Expression of at least some of these genes correlated with the *Trdv2-2* cluster c4, which expanded between days 21 and 42 (Fig. 2I). Collectively, our data suggest that type 3 intestinal Vγ6 cells undergo a rigorous immunological maturation during the first week of life, while their effector function is further induced during the weeks following weaning. Although similar molecular changes occur in Vγ4 cells, they additionally undergo type 3 programming after birth and type 1 programming, including cytotoxicity-related genes, after weaning.

### Tissue restricted adaptation of intestinal γδT17 cells

**Fig. 3.**
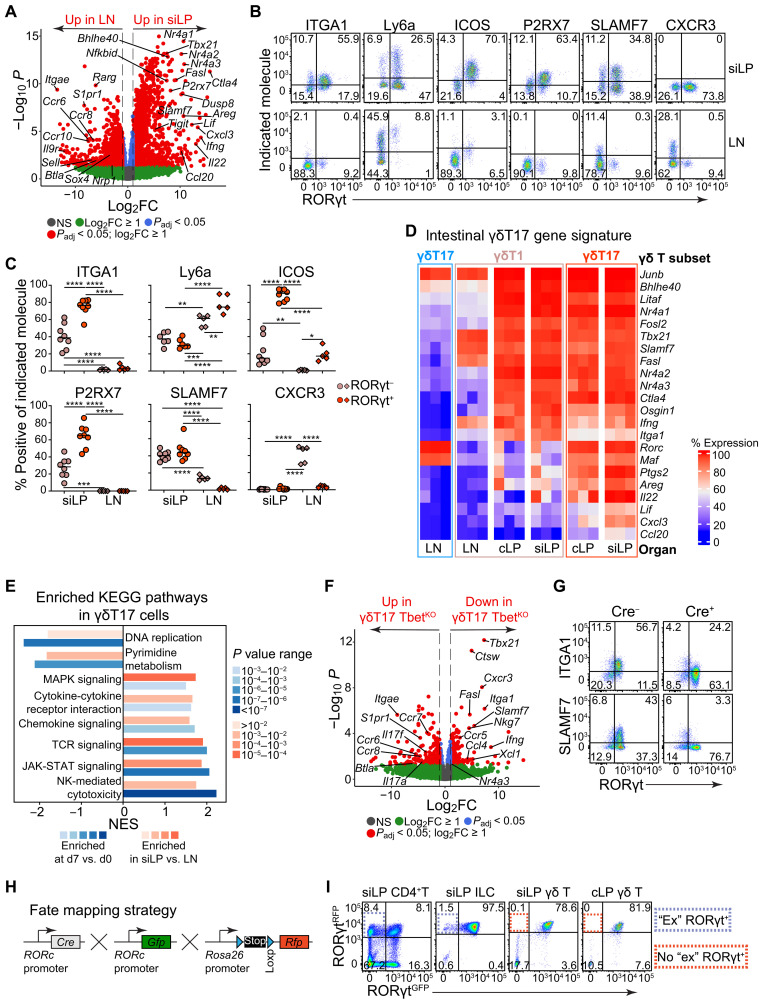
Tissue and Tbet-dependent imprinting of intestinal γδT17 cells. (**A**) Volcano plot indicating DEGs between siLP and LN γδT17 cells following bulk RNA-seq; selected genes enriched in either siLP or LN are indicated. (**B** and **C**) Representative plots (B) and quantification (C) of ITGA1, Ly6a, ICOS, P2RX7, SLAMF7, and CXCR3 levels in RORγt^+^ and RORγt^−^ γδ T cells from siLP or LN. (**D**) Heatmap showing the expression of the listed genes across γδ T cell subsets. (**E**) Enriched Kyoto Encyclopedia of Genes and Genomes (KEGG) pathways when comparing gut γδT17 cells from day 7 versus day 0 mice (blue) or adult siLP and LN γδT17 (orange). NES, normalized enrichment score. (**F**) Volcano plot indicating DEGs between Tbet-deficient (Tbet^KO^) and WT γδT17 cells from siLP; selected genes enriched in either Tbet^KO^ or WT are indicated. (**G**) Representative plots of SLAMF7 and ITGA1 levels in RORγt^+^ and RORγt^−^ γδ T cells from the siLP of Tbet^F/F^ (Cre^−^) or RORγt^CRE^-Tbet^F/F^ (Cre^+^) mice. (**H**) Schema of the genetic design used to lineage trace ex RORγt^+^ cells. (**I**) Representative analysis of the lineage tracing experiment indicating the expression of RORγt^GFP^ and RORγt^RFP^ in the indicated cells; ex RORγt^+^ cells express only RORγt^RFP^; representative of many mice over many different litters. In (A) and (D) to (F), three biological repeats, each consisting of a pool of four to six mice. In (A) and (F), each dot represents a gene. NS, not significant. In (B) and (C), three experiments, with five to eight mice. In (G), two experiments, with six mice. In (A), (D), and (F), *P* values were calculated using a quasi-likelihood ratio test with the R package edgeR and in (E) with R package fgsea v1.28.0. For (C), in all graphs, each symbol represents a mouse and line the median; **P < 0.01; ***P < 0.001; ****P < 0.0001, calculated using one-way ANOVA with Tukey’s multiple comparisons correction.

As the neonatal microenvironment in the gut is specialized in imprinting distinct immunological characteristics among cell lineages (*40*), we reasoned that intestinal LP γδ T cells would have considerably diversified from their LN counterparts. To test this, we used RORγt^GFP^Tbet^AmCyan^ mice to sort and perform bulk RNA-seq from cells residing in either the LN or the LP [both siLP and colonic LP (cLP)] that were of the γδT1 (RORγt^−^Tbet^+^) or γδT17 (RORγt^+^Tbet^+^ in LP and RORγt^+^Tbet^−^ in LN) subset (fig. S3A). Principal components analysis clustered the six populations based on cell type (γδT1 V γδT17) and organ (LN V gut). There was a clear diversification of LN from intestinal γδT17 cells, as well as from non-γδT17 populations (fig. S3B), suggesting a distinct organ-specific transcriptional identity. There were 2802 DEGs between siLP and LN γδT17 cells, of which 1416 were significantly higher in siLP and 1386 were significantly higher in LN (Fig. 3A and table S3). Similar differences were observed when comparing cLP LN γδT17, although the number of DEGs was considerably lower (1790 DEGs; 1140 higher in cLP and 650 higher in LN) (fig. S3C and table S3). The differences between siLP and cLP γδT17 cells amounted to only 454 genes, of which 206 were higher in siLP and 248 were higher in cLP (fig. S3D and table S3).

We noticed a number of immune-relevant genes that were highly and differentially expressed in the siLP compared to LN γδT17 cells, including cytokines (e.g., *Ifng* and *Il22*), chemokines (e.g., *Ccl20* and *Cxcl3*), transcription factors (e.g., *Tbx21* and *Bhlhe40*), immunoinhibitory receptors (e.g., *Ctla4* and *Tigit*), and effector (e.g., *Slamf7* and *Fasl*) and signaling molecules (e.g., *Dusp8* and *Nfkbid*) (Fig. 3A and table S3). LN cells expressed high levels of *S1pr1*, *Sell*, and *Ccr7*, reminiscent of their location, as well as the chemokine receptors *Ccr6* and *Ccr10* and the immunoregulatory receptors *Btla* and *Nrp1* (Fig. 3A and table S3). To validate the protein levels of some of the above genes, we decided to stain for surface molecules whose transcripts were highly expressed in adult intestinal γδT17 cells as well as some molecules that were detected in neonatal cells, which included CD49a (ITGA1), ICOS, CXCR3, Ly6a, P2RX7, and SLAMF7 (Fig. 3, B and C). RORγt^+^ cells in the gut differentially expressed high levels of ITGA1, ICOS, and P2RX7 when compared to either RORγt^−^ in the gut or all γδ T cells in the LN (Fig. 3, B and C). SLAMF7 was differentially expressed in the gut; however, both RORγt^+^ and RORγt^−^ cells had similar levels (Fig. 3, B and C). In contrast, levels of Ly6a were significantly higher in RORγt^+^ LN cells compared to all other subsets (Fig. 3, B and C). CXCR3 could only be detected in RORγt^−^ LN cells (Fig. 3, B and C). When we compared expression levels of these molecules in Vγ4^+^ and Vγ6^+^ intestinal RORγt^+^ γδ T cells, we found that ITGA1, P2RX7, and SLAMF7 were higher in Vγ6^+^, whereas Ly6a was higher in Vγ4^+^ cells (fig. S3E). In the LN, the only noticeable difference was the higher levels of ICOS in Vγ6^+^ cells (fig. S3E). Hence, besides Tbet, γδT17 cells in the gut also express ITGA1, SLAMF7, ICOS, and P2RX7.

Next, we wanted to define a gene signature that could discern intestinal γδT17 cells from their LN counterparts and also from intestinal γδT1 cells. Hence, we chose genes that were differentially expressed in the gut when compared to LN and also genes that were specific for the γδT17 lineage. We chose the following 22 genes: *Ctla4*, *Ifng*, *Ptgs2*, *Cxcl3*, *Lif*, *Il22*, *Nr4a1*, *Nr4a2*, *Nr4a3*, *Areg*, *Slamf7*, *Fasl*, *Itga1*, *Junb*, *Fosl2*, *Bhlhe40*, *Ccl20*, *Osgin1*, *Litaf*, *Tbx21*, *Rorc*, and *Maf* (Fig. 3D). It was obvious that in the gut, γδT17 and γδT1 cells shared a lot of these genes, pointing toward a substantial impact of the tissue and not lineage on transcriptional imprinting (Fig. 3D). For example, while all gut subsets expressed *Tbx21*, *Slamf7*, *Fasl*, and *Ifng*, only γδT1 cells did so in the LN (Fig. 3D). As a group, these genes were significantly more enriched in gut γδT1 than in LN γδT17 cells (fig. S3F), further suggesting a closer relationship between type 1 and type 3 γδ T cell lineages in the gut than between type 3 cells when these were residing at different tissues. Gene set enrichment analysis (GSEA) based on Kyoto Encyclopedia of Genes and Genomes (KEGG) pathways showed that the TCR, mitogen-activated protein kinase (MAPK), Janus kinase (JAK)–STAT, chemokine signaling, and cytokine-cytokine receptor interaction pathways were among the most highly enriched in siLP compared to LN γδT17 cells (Fig. 3E). GSEA analysis of our day 0 versus day 7 bulk RNA-seq dataset (fig. S2E) showed enrichment of the same pathways (Fig. 3E), substantiating our previous findings that the first week of life is a key period for γδ T cell maturation. Collectively, our data suggest that the gut microenvironment imprints γδ T cells with a tissue-specific phenotype and transcriptional profile that is initiated during early neonatal life, making gut γδT17 more similar to type 1 than to their type 3 counterparts in the LN.

### Tbet-dependent imprinting of intestinal γδT17 cells

We reasoned that one of the key drivers of the type 1 characteristics in gut γδT17 was Tbet. To test its role, we crossed RORγt-Cre with Tbet-flox (Tbet^F/F^) mice (RORγt^CRE^-Tbet^F/F^) and analyzed LP γδ T cells. Deletion of Tbet was confirmed by flow cytometry (fig. S4A). There was no difference in the numbers of total γδ T cells; however, there was a significant loss of Tbet^+^ γδ T cells in both siLP and cLP (fig. S4, B and C), whereas numbers of RORγt^+^ γδ T cells were unchanged (fig. S4, B and C). To get a better understanding about the impact of Tbet, we crossed Tbet^F/F^ with RORγt^CRE^-R26LSL^RFP^ mice, where red fluorescent protein (RFP) expression in the ROSA26 locus was induced by RORγt-driven Cre recombinase (fig. S4D). We sorted TCRγδ^+^RORγt^RFP^ (i.e. Tbet-deficient γδT17) cells from the siLP and cLP, performed bulk RNA-seq, and compared them to the gene sets we had generated earlier from Tbet-expressing cells. There were 504 DEGs between Tbet-deficient and Tbet-sufficient γδT17 cells in the siLP (Fig. 3F and table S3) and 595 in the cLP (fig. S4E and table S3). Many of the immune-relevant genes that were down-regulated in gut (siLP + cLP) Tbet-deficient cells included type 1 signature genes such as *Tbx21*, *Ifng*, *Ccr5*, *Cxcr3*, *Itga1*, *Ctsw*, *Ccl4*, *Xcl1*, *Nkg7*, and *Slamf7*, in addition to *Nr4a3*, *Nr4a1*, and *Fasl*, while *Il17a* and *Il17f* were significantly up-regulated (Fig. 3F and fig. S4E). We confirmed Tbet-dependent expression of ITGA1 and SLAMF7 by flow cytometry in both Vγ4^+^ and Vγ6^+^ cells (Fig. 3G and fig. S4, F and G). In contrast, some of the genes that were up-regulated in Tbet-deficient cells were also highly enriched in LN γδT17, such as *S1pr1*, *Ccr7*, *Ccr6*, *Ccr8*, *Ccr10*, *Itgae*, *Nrp1*, and *Btla* (Fig. 3, A and F). Thus, Tbet promotes type 1 imprinting of intestinal γδT17 cells.

It was plausible that Tbet marked type 3 to type 1 plasticity, so that, at some point, intestinal γδT17 cells would lose RORγt expression and acquire a Tbet-only identity, similar to group 3 innate lymphoid cells (ILC3) (*41*, *42*). To test this, we performed a lineage tracing experiment by crossing RORγt^GFP^ to RORγt^CRE^-R26LSL^RFP^ mice (Fig. 3H). As opposed to CD4^+^ T cells and ILC3, we could not detect any GFP^−^RFP^+^ “ex” RORγt^+^ γδ T cells (Fig. 3I), in agreement with previously published data (*43*). This suggested that at steady-state and in the absence of immunogenic and activation signals, although intestinal γδT17 cells express Tbet and acquire a type 1 program, they do not lose their type 3 identity.

**Fig. 4.**
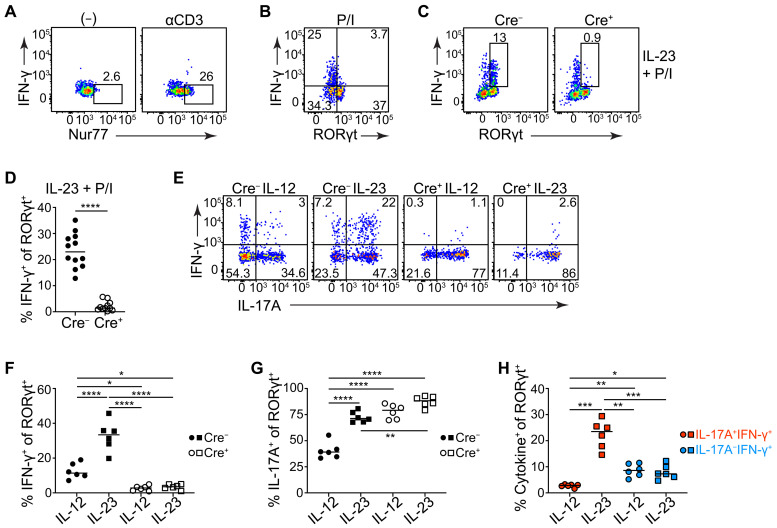
Tbet differentially regulates IFN-γ and IL-17A production. (**A** and **B**) Representative flow cytometry analysis of IFN-γ production in siLP RORγt^+^ γδ T cells stimulated with αCD3 or PMA + ionomycin (P/I); in (A), cells are pregated as RORγt^+^; (three experiments, with 11 mice); in (B), two experiments, with seven mice. (**C** and **D**) Representative plots (C) and quantification (D) of IFN-γ levels in siLP RORγt^+^ γδ T cells after stimulation with IL-23 + PMA + ionomycin from Tbet^F/F^ (Cre^−^) or RORγt^CRE^-Tbet^F/F^ (Cre^+^) mice; in (D), cells are pregated as RORγt^+^ (four experiments, with 11 to 12 mice). (**E**) Representative plots of IFN-γ and IL-17A production in Cre^−^ and Cre^+^ siLP RORγt^+^ γδ T cells after stimulation with IL-23 or IL-12. (**F** and **G**) Quantification of IFN-γ and IL-17A levels in Cre^−^ and Cre^+^ siLP RORγt^+^ γδ T cells after stimulation with IL-23 or IL-12. (**H**) Frequencies of IL-17A^+^IFN-γ^+^ (red) and IL-17A^−^IFN-γ^+^ (blue) RORγt^+^ γδ T cells after stimulation with IL-23 or IL-12. In (E) to (H), two experiments, with six mice. In all graphs, each symbol represents a mouse and line the median; *P < 0.05; **P < 0.01; ***P < 0.001; ****P < 0.0001 calculated using the Mann-Whitney test in (D) and a one-way ANOVA with Tukey’s multiple-comparisons correction in (F) to (H).

A key functional feature of type 1 immune cells is the production of IFN-γ, which is regulated by Tbet. We therefore tested whether this was also the case in γδT17 cells. To induce IFN-γ, we first activated siLP cells with anti-CD3 (αCD3) or a combination of phorbol 12-myristate 13-acetate (PMA) and ionomycin but could only measure negligible IFN-γ levels (Fig. 4, A and B). However, IFN-γ was induced in a Tbet-dependent manner when PMA and ionomycin were combined with IL-23 (Fig. 4, C and D). We then compared IL-23 and IL-12 stimulations, as the latter is a known type 1–inducing cytokine. We found that IFN-γ could be induced more potently by IL-23 than by IL-12 (Fig. 4, E and F) and, as expected, IL-23 was also better at inducing IL-17A and IL-17F (Fig. 4, E and G, and fig. S5, A and B). IL-23 was particularly effective at supporting cells coproducing IFN-γ with IL-17A and IL-17F (Fig. 4, E and H, and fig. S5, A and C). Frequencies of IFN-γ^+^IL-17A^−^ cells were similar between IL-12 and IL-23 (Fig. 4H), while frequencies of IFN-γ^+^IL-17F^−^ cells were only slightly higher following IL-23 stimulation (fig. S5C). Notably, lack of Tbet resulted in high proportions of cells producing IL-17A or IL-17F (Fig. 4, E and G, and fig. S5, A and B), in agreement with the high *Il17a* and *Il17f* expression levels in Tbet-deficient cells (Fig. 3F). IL-22 could not be induced by IL-12 and was Tbet independent (fig. S5D). In summary, these data show that Tbet promotes type 1 and suppresses type 3 functional properties in intestinal γδT17 cells.

### Heightened TCR signaling capacity in intestinal γδT17 cells

**Fig. 5.**
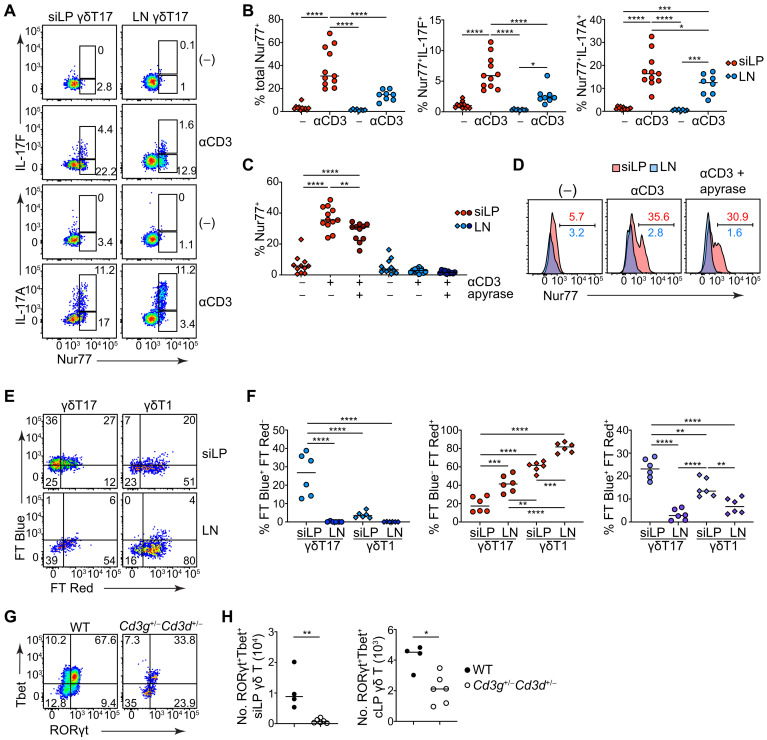
Heightened TCR signaling capacity in intestinal γδT17 cells. (**A** and **B**) Representative plots (A) and quantification (B) of Nur77 expression and IL-17A/F production in siLP and LN γδT17 cells after a 3-hour culture with or without αCD3 [three experiments, with 11 mice (siLP) or 8 mice (LN)]; in (B) (left plot), total Nur77^+^ cells are the addition of Nur77^+^Cytokine^+^ and Nur77^+^Cytokine^−^ cells shown in the flow cytometry plots in (A). (**C** and **D**) Quantification (C) and representative histograms (D) of Nur77 expression in siLP and LN γδT17 cells after a 3-hour stimulation under the indicated conditions (four experiments, with 10 to 12 mice). (**E** and **F**) Representative plots (E) and quantification (F) of FT Blue^+^ (early Nur77 activation), FT Red^+^ (late Nur77 activation) and FT Blue^+^Red^+^ in siLP and LN γδT17 cells isolated from Tempo mice (two experiments, with six mice). (**G** and **H**) Representative plots (G) and quantification (H) of RORγt^+^Tbet^+^ γδ T cells in the siLP (G) and siLP or cLP (H) of WT and CD3g^+/-^CD3d^+/−^ mice (two experiments, with four or six mice). In all graphs, each symbol represents a mouse and line the median; *P < 0.05; **P < 0.01; ***P < 0.001; ****P < 0.0001 using one-way ANOVA with Tukey’s multiple comparisons correction in (B), (C), and (F) or the Mann-Whitney test in (H).

Contrary to their need for TCR signaling during thymic development, adult γδT17 cells in the LN have been shown to be hypo-responsive to TCR stimuli (*44*). However, our earlier observation that the TCR contributes to the expression of Tbet (see Fig. 1F) made us hypothesize that in the gut, where Tbet expression is evident, the γδT17 TCR cells may be more active. This was reinforced by the fact that TCR signaling was one the most highly enriched pathways in day 7 and adult intestinal γδT17 cells (Fig. 3E) and that some of the most highly expressed genes were those coding the orphan nuclear receptors *Nr4a1*, *Nr4a2*, and *Nr4a3* (Fig. 3A), which function as transcription factors and can be directly induced by the TCR (*45*). To test whether intestinal γδT17 cells had increased TCR signaling capacity, we activated LN and siLP lymphocytes with αCD3 in the absence of other stimuli and assessed expression of Nur77 (*Nr4a1*) and production of IL-17A and IL-17F 3 hours later. We found that Nur77 was induced at higher levels in the siLP than the LN and that Nur77^+^ cells expressed both IL-17A and IL-17F (Fig. 5, A and B). Production of IL-17A and IL-17F was significantly higher in the siLP following αCD3 stimulation (Fig. 5B). Induction of Nur77 in the gut was similar between Vγ4^+^ and Vγ6^+^ cells (fig. S6A). We reasoned that the higher activity of the TCR in the gut could be attributed to the presence of the microbiota. However, induction of Nur77 was similar between GF and specific pathogen-free (SPF) mice (fig. S6B). Since P2RX7 was highly expressed in intestinal γδT17 cells and this receptor has been shown to amplify TCR-mediated Ca^2+^ signaling through adenosine 5′-triphosphate (ATP) (*46*), we reasoned that it might be involved in determining the higher TCR reactivity of intestinal γδT17 cells. To test this, we repeated the above experiments in the presence of apyrase, which hydrolyses and depletes ATP, and found that it reduced significantly the induction of Nur77 following αCD3 stimulation (Fig. 5, C and D). However, induction of Nur77 in siLP cells deficient for P2RX7 was not changed (fig. S6C). Thus, in intestinal γδT17 cells, TCR stimulation leads to functional signaling.

Next, we wanted to test whether intestinal γδT17 cells are actively engaging their TCR in situ. To directly test this, we used Nur77–timer rapidly expressed in lymphocytes (Tempo) mice, which report a fluorescent timer (FT) protein under the control of Nur77 (*47*). In these mice, a FT Blue signal denotes recent TCR-driven Nur77 expression, while FT conversion to a Red signal indicates accumulated past signaling (*47*). We found that siLP γδT17 cells had significantly higher levels of FT Blue and FT Blue/Red signal compared to their LN counterparts suggesting active and recent TCR activation (Fig. 5, E and F). In contrast, LN γδT17 and γδT1 cells had higher levels of FT Red suggestive of past TCR signaling (Fig. 5, E and F). Last, we investigated intestinal γδT17 cells in the CD3 double haploinsufficient *CD3g*^+/−^*CD3d*^+/−^ mice, which have impaired TCRγδ signaling and γδ T cell subset development (*5*). Numbers of RORγt^+^Tbet^+^ γδ T cells in the gut were significantly reduced in these mice, further supporting the importance of the TCR (Fig. 5, G and H); however, this may reflect signaling requirements in the thymus and not TCR-driven homeostasis or maintenance in the tissue. In summary, these data suggest that in the gut, γδT17 cells actively engage their TCR resulting in heightened signaling capacity, which can be sufficient to induce cytokine production and which is partly dependent on ATP.

### Presence of type 3 γδ T cells in the human neonatal intestine

**Fig. 6.**
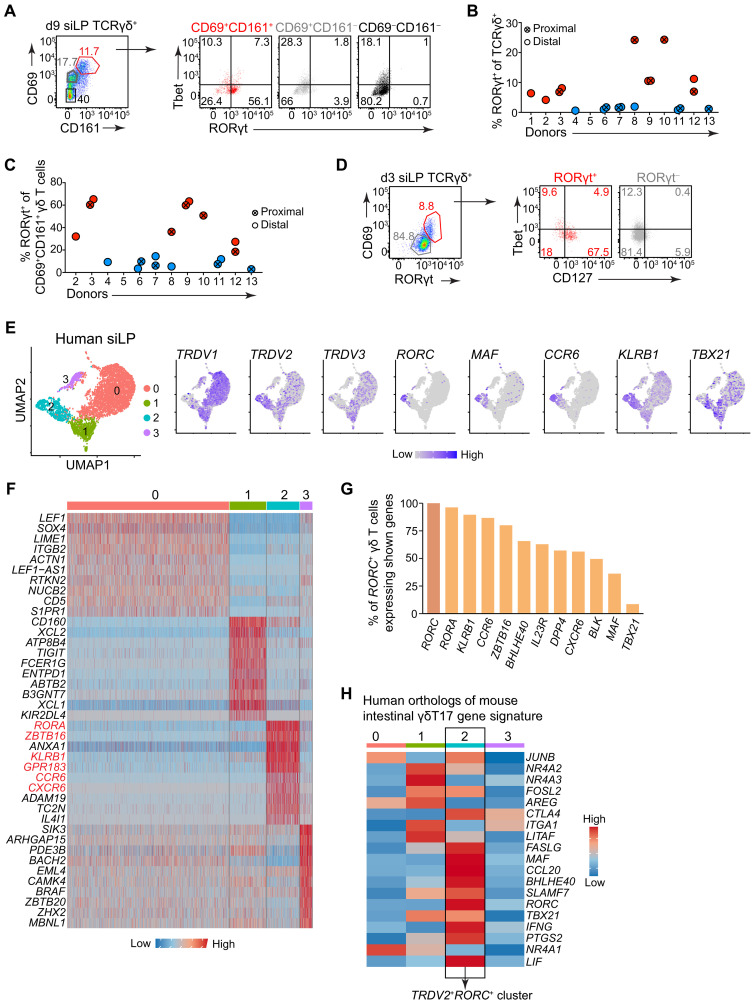
Presence of RORγt-expressing type 3 γδ T cells in the human neonatal intestine. (**A**) Representative flow cytometry analysis of human neonatal siLP from donor 9 (d9) indicating the presence of CD69^+^CD161^+^, CD69^+^CD161^−^, and CD69^−^CD161^−^ γδ T cells and their expression of RORγt and Tbet. (**B**) Quantification of RORγt^+^ γδ T cells across all donors. (**C**) Quantification of RORγt^+^ cells within CD69^+^CD161^+^ γδ T cells across all donors (except donor 1). [(B) and (C)] Red, detectable RORγt^+^ cells; blue, not detectable RORγt^+^ cells. (**D**) Representative flow cytometry analysis of human neonatal siLP (donor 3) indicating the presence of RORγt^+^CD69^+^ and RORγt^−^CD69^−^ γδ T cells and their expression of CD127 and Tbet. (**E**) UMAP showing how γδ T cell populations cluster in the human neonatal siLP and feature plots showing log-normalized expression levels of *TRDV1*, *TRDV2*, *TRDV3*, *RORC*, *MAF*, *CCR6*, *KLRB1*, and *TBX21* among these clusters. (**F**) Heatmap showing the top 10 DEGs for each γδ T cell cluster in the human neonatal siLP; all shown genes were significantly expressed for each cluster (*P* < 0.05; calculated using the Wilcoxon rank sum test) with a cutoff expression of log_2_FC of ≥1; gene names in red indicate relevance to type 3 immunity. (**G**) Graphical representation of the frequency of *RORC*^+^ cells expressing the indicated genes in human neonatal siLP; bars indicate the percentage of *RORC*-expressing cells with measurable (>0 counts) expression of each gene; genes are ordered by decreasing prevalence. (**H**) Heatmap showing, for each cluster, the expression level of the human ortholog genes of the mouse intestinal γδT17 signature that we defined in Fig. 3D.

Last, we explored whether the human gut contained γδ T cells resembling mouse intestinal γδT17. As there is evidence suggesting that neonatal blood and thymus in humans are enriched in type 3 γδ T cells (*33*, *34*), we analyzed human neonatal siLP and cLP from 13 infants who were undergoing either primary or secondary (anastomosis) surgery for necrotizing enterocolitis (NEC) or unrelated noninflammatory conditions (Table 1). To test for the presence of putative γδT17 populations, we considered cells that were CD19^−^CD14^−^CD11b^−^HLA-DR^−^CD4^−^CD45^+^CD3^+^TCRγδ^+^ and expressed or not RORγt. We found that RORγt^+^ cells were present in varying frequencies but not in all donors (Fig. 6, A and B, and fig. S7, A and B) and, in one case, only in the proximal but not distal part of the resected tissue (donor 8 in Fig. 6, B and C). However, when present, RORγt^+^ cells were always CD69^+^CD161^+^ (Fig. 6, A and C), and they expressed CD127 (IL-7Rα) but no detectable Tbet (Fig. 6, A and D). A CD69^+^CD161^+^ population was evident in all donors (fig. S7C).

**Table 1.** Clinical information of neonatal donors with intestinal disorders. For each donor, it is listed diagnosis, gestational age (GA), type of surgery at sample collection [primary (1) or secondary (2)], and postnatal age (age) when the sample was collected (d, days; m, months). Term status was defined on the basis of the corrected age (gestational age plus postnatal age at the time of sample collection) and classified as preterm (<37 weeks), term (37 to 42 weeks), or postterm (>42 weeks). SI, small intestine.

Donor	Diagnosis	GA (week)	Surgery	Age	Term status
1	Meconium plug at ileocecal site	24	2	3 m	Term
2	Perforation in colon descendant	31	2	2 m	Term
3	SI atresia	39	2	1 m	Postterm
4	Resection of rectum and sigmoid colon	38	1	2 m	Postterm
5	Colon atresia	40	1	2d	Term
6	NEC	28	2	2 m	Preterm
7	Other	33	1	27d	Term
8	NEC	27	2	4 m	Postterm
9	SI atresia	37	1	1d	Term
10	NEC	28	1	12d	Preterm
11	SI atresia	38	1	1d	Term
12	Other	27	2	2 m	Term
13	SI atresia	33	1	1d	Preterm

To get a more unbiased and detailed overview of γδ T cells in the human neonatal LP, we performed scRNA-seq from sorted TCRγδ^+^ cells derived from two different donors, one siLP and one cLP. We identified four clusters in the siLP (Fig. 6E and table S4) and five clusters in the cLP (fig. S7D and table S4), which contained all the three major γδ T cell populations based on the expression of *TRDV1* (Vδ1^+^), *TRDV2* (Vδ2^+^), and *TRDV3* (Vδ3^+^) (Fig. 6E and fig. S7D). Whereas Vδ1^+^ cells were distributed among most clusters, Vδ2^+^ cells were mostly constrained within cluster c2 in both siLP and cLP (Fig. 6E and fig. S7D). Vδ3^+^ cells were less frequent and had a similar distribution to Vδ1^+^ cells (Fig. 6E and fig. S7D). We then examined the top DEGs among the clusters (table S4) in an attempt to identify groups of genes that may reveal putative functions, in particular those related to type 3 immunity. In this regard, we observed that cluster c2 in both siLP (Fig. 6F) and cLP (fig. S7E) expressed genes previously related with type 3 immune cells including *RORA*, *KLRB1* (CD161), *CCR6*, *ZBTB16*, and *CXCR6* (*25*), as well as *GPR183* (Fig. 6F and fig. S7E), which is linked to mouse γδT17 cell development and function (*48*).

Next, we looked for *RORC*-expressing cells and could find them concentrated in cluster c2 in both siLP and cLP with few cells also present in cluster c3 (Fig. 6E and fig. S7D). *RORC*^+^ cells colocalized with cells expressing the type 3–associated genes *MAF*, *CCR6*, and *KLRB1*, while coexpression with *TBX21* was weaker (Fig. 6E and fig. S7D). We then focused on *RORC*^+^ cells and the top genes that they expressed in siLP and cLP and identified many that have been previously described as being signature γδT17 or type 3 genes in both mouse and human (fig. S8, A and B). We selected some of these type 3 genes (*MAF*, *CCR6*, *KLRB1*, *DPP4*, *RORA*, *IL23R*, *CXCR6*, *BLK*, *BHLHE40*, and *ZBTB16*) together with *TBX21* and plotted their frequency of expression in *RORC*^+^ cells (Fig. 6G and fig. S7F). We found that to a varying degree, human gut neonatal *RORC*^+^ γδ T cells expressed all the above type 3 signature genes in addition to *TBX21*, albeit the latter at low levels (Fig. 6G and fig. S7F). Many human orthologs of the genes that comprised the mouse intestinal γδT17 signature (Fig. 3D) were also expressed at medium or high levels by *RORC*^+^ cluster c2 cells (Fig. 6H and fig. S7G). Collectively, these data suggest that the human neonatal intestine contains γδ T cells with type 3 characteristics closely resembling mouse intestinal γδT17 cells.

## DISCUSSION

In the present study, we show that γδ T cells localize to the gut in embryonic and neonatal life, during which they begin expressing Tbet most likely in response to cytokine and TCR activation signals. Both γδT17 and γδT1 populations undergo temporal immunological maturation from birth to adulthood, including type 3 and type 1 programming, with the first week of life and the postweaning period being key time points in this process. Subsequently, γδT17 cells become imprinted with type 1 properties in a Tbet-dependent manner and assume a distinct transcriptional, phenotypical, and functional profile. Compared to cells in the LN, intestinal γδT17 cells had more active TCR signaling that was partly dependent on ATP and was sufficient to induce cytokine production (fig. S9). We also provide evidence that the human neonatal gut contains γδ T cells that are RORγt^+^CD69^+^CD161^+^ and share a transcriptional profile similar to mouse intestinal γδT17 cells.

The cellular and molecular changes that occur during neonatal life together with bacterial colonization and introduction of diet imprint the immune system permanently and irreversibly (*27*, *40*, *49*). There is evidence that many innate and adaptive immune cell subsets undergo extensive immunological maturation and functional alterations during neonatal life (*50*). Moreover, there are specialized neonatal αβ T and B cells, which are tailored for that specific time period and which persist to adult life (*51*, *52*). As innate γδ T cells develop their basic functional properties in the thymus, they will enter neonatal tissues to encounter a rapidly changing environment, with which they interact and receive signals from. For example, butyrophilin-like molecules in mice shape the maturation of intraepithelial Vγ7^+^ cells during neonatal life, and this is independent of the microbiota or diet (*30*). The data presented herein provide evidence that in the intestinal LP, γδ T cells have a biphasic response to the neonatal microenvironment, one during the first week of life characterized by type 3 programming and immunological maturation and one after weaning characterized by type 1 programming and enhanced effector function. These neonatal and organ-specific imprinting diversified intestinal type 3 populations instilling distinct transcriptional and phenotypical properties. Thus, neonatal life is a fundamental period for innate γδ T cells, and remains to be seen what are the sensors and triggers that imprint their biology. This notwithstanding, their vigorous and rapid tissue adaptation in early life may help explain their acquisition of specialized functions within organs (*11*–*13*).

A hallmark of intestinal type 3 γδ T cells is their expression of Tbet, which guides their transition toward a type 1 effector program, while, at the same time, inhibiting IL-17 production. The importance of Tbet as a master transcription factor for type 1 T cells is undisputed (*53*), while it is also well documented that it can modulate the development and function of both innate and adaptive type 3 lymphocytes (*54*, *55*), although this appears to be cell and model specific. Thus, in ILC3, Tbet induces their differentiation into natural cytotoxicity triggering receptor 1 (NKp46^+^)IFN-γ–producing cells important for clearing intestinal infections (*54*), whereas in T helper 17 (T_H_17) cells, it is more important for their transition into “ex T_H_17” T_H_1 cells rather than IFN-γ production (*55*–*57*). Furthermore, Tbet was not required for pathogenesis in the experimental autoimmune encephalomyelitis or *Helicobacter hepaticus–*induced intestinal inflammation models (*56*, *57*), and its absence correlated with high amounts of IL-17 production in the cell transfer colitis model (*55*). The data herein suggest that the importance of Tbet in γδ T cells is reminiscent of both innate and adaptive lymphocytes. Thus, IFN-γ production was strictly Tbet dependent as in ILC3 (*54*), but its induction was more IL-23 dependent as in T_H_ cells (*56*). Furthermore, Tbet suppressed IL-17 production similar to T_H_ cells but not ILC3 (*54*, *55*). Ultimately, Tbet generated an ITGA1^+^SLAMF7^+^RORγt^+^ intestinal γδ T cell population with a clear type 1 transcriptional and functional makeup that diversified from other type 3 γδ T cells. RORγt^+^Tbet^+^ intestinal γδ T cells do not transition into “ex RORγt” like ILC3 or T_H_17 at steady state; however, in the context of infection, they can switch off IL-17 to become IFN-γ–producing-only, a process that is regulated by the activating protein 1 family of transcription factors (*43*). Whether γδT17 plasticity is also imprinted by their specific tissue microenvironment remains to be elucidated.

A key determinant of the “innateness” of γδT17 and other γδ T cell populations is their ability to produce effector molecules and to proliferate in response to cytokines only, without the need for TCR engagement (*15*). It has been shown that innate type 1 and type 3 γδ T cells from the LN are very hyporesponsive to TCR stimulation (*44*). This is in marked contrast with their requirement for active TCR signaling, albeit weak, during thymic development (*3*, *5*, *6*). Hence, it appears that as they develop, innate γδ T cells attenuate their TCR responsiveness. However, in the gut, γδT17 cells had an active and responsive TCR whose engagement, at least ex vivo, was sufficient to induce IL-17 but not IFN-γ. This is in line with dermal Vγ6^+^ γδT17 cells that had constitutive expression of *Nr4a1* and its protein product Nur77 (*58*). Our transcriptional data are suggestive that the TCR develops the propensity for activation during the first week of life. Whether this is the default mechanism for the gut and other nonlymphoid tissues and when cells end up in the LN miss a TCR activation signal is not known. It is also not known whether increased TCR activity at barrier tissues, such as the gut and skin, is due to the presence of high concentrations of specific ligands that are absent from or at low levels in LNs. In this regard, we found that both Vγ4 and Vγ6 TCRs were active in the gut, indicating the presence of ligands for both receptors, or that there is TCR promiscuity for the same ligand(s). These putative ligands could function in a similar way as butyrophilin-like molecules for Vγ5 and Vγ7 cells and select and/or sustain γδT17 cells in the LP. Alternatively, the TCRs remain minimally engaged in all organs, but other factors promote a stronger signaling in the gut and skin. To this end, we found that the extracellular ATP sensor P2RX7 and the costimulatory receptor ICOS, both of which can enhance TCR signaling (*46*, *59*), were highly and differentially expressed in the gut providing a plausible explanation for the increased TCR activity. Depletion of ATP reduced intestinal TCR responses; however, P2RX7 deficiency did not have any effect. Combined, the data suggest that in the gut, innate γδT17 cells have a responsive TCR, which can be sufficient to activate them and induce production of cytokines.

The presence of bona fide γδT17 cell populations in humans has been in doubt and with contradictory data. For example, although clearly present in the psoriatic skin (*8*, *21*), they are absent from the healthy skin (*19*). Similarly, a study of a cohort of patients with colon carcinoma showed plentiful γδT17 cells in the diseased gut (*20*); however, a study of a different patient cohort did not find evidence for IL-17 production by gut γδ T cells (*60*). Whereas human IL-17–producing γδ T cells are very rare in the adult blood (*17*, *18*), they are more prevalent in the cord blood (*33*, *34*). Moreover, scRNA-seq studies in the neonatal cord blood and human embryonic thymus showed the presence of RORγt-expressing γδ T cells with a type 3 transcriptome that delineates them from other γδ T cell subsets (*17*, *25*). The team of Vermijlen (*25*) additionally found that the embryonic human thymus contains a Vδ2^+^ cell with a hybrid type 1/3 transcriptional profile. Here, we report the presence of type 3 γδ T cells in the human neonatal gut. When present, these neonatal cells were always RORγt^+^CD161^+^CD69^+^, expressed CD127 (IL-7Rα), and they could be derived from both the small intestine and colon. Moreover, scRNA-seq data from two donors showed the presence of γδ T cells coexpressing transcripts for Vδ2, RORγt, and many type 3–associated molecules, as well as genes we identified in mouse intestinal γδT17 cells. However, expression of Tbet could only be detected at the transcriptional level and at low levels, which could be explained by the young age of the infants we studied. Alternatively, besides an apparent evolutionary conservation between mouse and human innate type 3 γδ T cells in the gut, there are specific fundamental differences. We cannot exclude, however, that interdonor variability and clinical context, such as inflammatory status and gestational age, contributed to these results.

Collectively, innate γδ T cell populations in the intestinal LP undergo intensive immunological maturation during neonatal life, resulting in the imprinting of distinct transcriptional, phenotypical, and functional characteristics that merge type 1 and type 3 lineages to generate cells specific to their microenvironment. It would be intriguing to test whether neonatal acquisition of tissue residency is a common process across organs.

## MATERIALS AND METHODS

### Mice

With the exception of CD3 double-haploinsufficient (CD3DH), P2RX7^−/−^, and Tempo mice, all other mouse strains were bred and maintained in-house at the Technical University of Denmark BioFacility under the breeding license 2024-15-0202-00220 approved by the Danish Animal Experiments Inspectorate. All experiments with mice were performed with the approval of the Danish Animal Experiments Inspectorate under the licenses 2019-15-0201-00385 and 2022-15-0201-01171. RORγt^CRE^ mice were provided by G. Eberl (Pasteur Institute, Paris, France) (*61*). Tbet^F/F^ mice were provided by B. J. Lindbom (Lund University, Lund, Sweden). RORγt^GFP^Tbet^AmCyan^ mice were previously described (*28*). ROSA26-floxSTOPflox-RFP mice were from the Swiss Immunological Mouse Repository. For fate mapping experiments, RORγt^CRE^-R26LSL^RFP^ were crossed to RORγt^GFP^Tbet^AmCyan^ mice. CD3DH mice were provided by N. Gonçalves-Sousa and B. Silva-Santos (Instituto de Medicina Molecular, Universidade de Lisboa, Portugal) (*5*). P2RX7^−/−^ mice were provided by A. Nicke and J. D. S. Marquez (Ludwig-Maximilians-Universtät, München). Tempo mice were generated as previously described (*47*), and experiments were performed at the University of Birmingham, approved by the local animal welfare and ethical review body, and authorized under the authority of the Home Office (*62*). GF mice were generated and maintained at the National Food Institute, Technical University of Denmark. GF status was confirmed by inoculation of feces from all mice into brain heart infusion (BHI) broth (25° and 37°C, aerobic incubation), modified gifu anaerobic medium (GAM) broth (37°C, anaerobic incubation), and plating on blood agar (37°C, aerobic incubation) and evaluation after 24 hours and 2 weeks of incubation.

### Human tissue

As reported in (*62*), neonatal human tissue was obtained from the Department of Neonatal and Pediatric Intensive Care and Department of Pediatric Surgery at Rigshospitalet, Copenhagen under the ethical permit S-20220057 approved by the Ethical Committee of Southern Denmark. Tissue was donated after verbal consent and collected at primary or secondary surgery. Donor and sample information are detailed in Table 1.

### Cell culture media and buffers

All cell cultures and single-cell suspensions were prepared using RPMI 1640 (Invitrogen) supplemented with 10% heat-inactivated fetal bovine serum (FBS; Gibco), 20 mM Hepes (pH 7.4) (Gibco), 50 μM 2-mercaptoethanol, 2 mM l-glutamine (Gibco), and penicillin-streptomycin (p/s; 100 U/ml; Gibco).

Hanks’ balanced salt solution (HBSS; 10×; Gibco) was diluted to 1× with sterile Milli-Q water and supplemented with 15 mM Hepes to prepare HBSS-Hepes or supplemented with 2 mM EDTA (Gibco), 15 mM Hepes, gentamycin (50 μg/ml; Gibco), p/s (100 U/ml), and 2% FBS to prepare HBSS-EDTA. Isotonic Percoll was prepared by mixing 90% Percoll (GE Healthcare) with 9% 10× HBSS and 1% Hepes and then diluted with HBSS-EDTA to the desired concentration. Human tissue was washed in R5 buffer [RPMI 1640 supplemented with 5% heat-inactivated FBS and p/s (100 U/ml)]. Dithiothreitol (DTT) buffer was prepared by mixing R5 buffer with DTT (4 μl/ml; Roche). Fluorescence-activated cell sorting (FACS) buffer was prepared by supplementing Dulbecco’s phosphate-buffered saline (DPBS; Thermo Fisher Scientific) with 3% heat-inactivated FBS. Magnetic-activated cell sorting (MACS) buffer was prepared by supplementing DPBS with 2% heat-inactivated FBS and 1 mM EDTA (*62*).

### *C. albicans* strain and infection model

For infections, we used *C. albicans* strain 101 (*63*) grown in yeast extract, peptone, and dextrose (YPD) medium at 30°C and 250 rpm for 16 to 18 hours. Pregnant female mice were treated with a mix of streptomycin (5 mg/ml) and ampicillin (1 mg/ml) (both from Sigma-Aldrich) in their drinking water supplemented with 2% sucrose, starting 1 week before delivery and continuing until the pups were euthanized. The antibiotic-containing water was replaced every 2 to 3 days. Six- to 10-day-old pups of these females were infected by oral gavage with 3.5 × 10^5^ to 4.7 × 10^5^ *C. albicans* yeast cells and euthanized 4 days postinfection. For determination of the fungal burden, the small intestine and colon were collected in cold PBS, cleaned, and homogenized in sterile 0.05% NP-40 for 3 min at 25 Hz using a TissueLyser (QIAGEN). Homogenates were plated on YPD agar containing penicillin (100 U/ml) and streptomycin (100 μg/ml) and incubated at 37°C for 2 days.

### Lymphocyte isolation from mouse organs

Peripheral LNs (axial, brachial, and inguinal) from adult mice and thymic lobes from E18.5 or newborn mice were dissected, cleared of fat, and crushed against a 70-μm cell strainer. Cell suspensions were then washed and filtered through a 40-μm cell strainer. For siLP and cLP lymphocytes, small intestines and colons were dissected from adult mice and were flushed with 20 ml of HBSS-Hepes to remove intestinal contents. Fat and Peyer’s patches were removed, and the tissues were opened longitudinally and cut into small pieces of ∼1 cm. Tissue was washed four times in HBSS-EDTA buffer at 37°C while shaking at 400 rpm and then digested using Liberase (60 μg/ml; Roche) and deoxyribonuclease (DNase; 40 μg/ml; Sigma-Aldrich) per sample in supplemented RPMI 1640 for 45 min at 37°C on a magnetic stirrer (800 rpm). The resulting cell suspensions were filtered through 70-μm cell strainers and washed once in supplemented RPMI 1640. Lymphocytes were separated on a 40/70 Percoll gradient. Cells from the interphase were collected and washed once before use. For neonatal gut samples, the Percoll gradient was omitted. For embryonic gut samples, tissue was digested immediately after dissection, and cell suspensions were then filtered through 40-μm cell strainer, washed, and used in downstream applications (*62*).

### Ex vivo culturing of lymphocytes

For staining of cytokines from lymphocytes isolated from LP, cell suspensions were stimulated with IL-23 (40 ng/ml) or IL-12 (20 ng/ml) (R&D Systems) for 3 hours, followed by PMA (50 ng/ml; Sigma-Aldrich), ionomycin (750 ng/ml; Sigma-Aldrich), and BD GolgiStop (1 μl/ml; containing monensin) for an additional 3.5 hours. For detection of IL-17A/F production in Nur77^+^ cells, peripheral LN lymphocytes (5 × 10^6^ cells/ml) or siLP suspensions were cultured for 3 hours in a precoated plate with αCD3 (5 μg/ml; clone 145-2C11), and BD GolgiStop (1 μl/ml) was added to all conditions. When indicated, apyrase (10 U/ml) was also added. Thymocytes (2 × 10^6^ cells/ml) from newborn mice were cultured for 48 hours with IL-7 (20 ng/ml), IL-23 (40 ng/ml), IL-1β (20 ng/ml), or their combinations, in the presence or absence of GL3-coated plate (1 μg/ml). In all cases, after incubations, the cells were harvested and used for flow cytometry staining (*62*).

### Transfer in RAG1^−/−^ mice and treatment with FTY720

Thymocytes from newborn (0- to 1-day-old) RORγt^GFP^Tbet^AmCyan^ mice were prepared and transferred into RAG1^−/−^ hosts. The procedure has been described in detail before (*29*). Reconstitution of γδ T cells in intestinal LP was analyzed 6 weeks after transfer in neonatal recipients and 3 weeks after transfer in adult RAG1^−/−^ recipients. For FTY720 treatments, neonatal mice were group weighed immediately after birth and injected intraperitoneally with 50 μl of FTY720 (1 mg/kg; Cayman Chemical). Injections continued once daily for 1 week (*62*).

### Lymphocyte isolation from human tissue

After washing in R5 buffer, removing visible fat, and dissecting out muscle under a stereoscope, the gut tissue was rinsed with DTT buffer twice for 10 min at 37°C in a shaking incubator (320 rpm), followed by four washes with HBSS-EDTA buffer at 37°C (320 rpm, 10 min each wash). Tissue was cut in small pieces (1 to 2 mm^2^) and digested using collagenase D (25 μl/ml; Roche) and DNase (50 U/ml; Roche) on a magnetic stirrer (800 rpm) for 45 min at 37°C. LP was subsequently dissociated with a Pasteur pipette and crushed against a 70-μm cell strainer. Cell suspensions were washed, filtered through a 40-μm cell strainer, and used for experiments (*62*).

### Flow cytometry staining

As reported in (*62*), both mouse and human cells were stained with Fixable Viability Stain 700 (1:1000; BD Horizon) or Fixable Viability Dye eFluor 780 (1:2000; Invitrogen) in PBS for 10 min on ice. Surface antigens, intracellular cytokines, and transcription factors were stained for flow cytometry as previously described (*29*). Cells were acquired using BD LSRFortessa or BD FACSymphony A5 SE for spectral acquisitions, and data were analyzed using FlowJo version 10.8.0 software (BD Biosciences). The following antibodies were used herein at 1:200 dilution, unless otherwise specified:

1) Mouse: anti-CD19 [6D5; fluorescein isothiocyanate (FITC)], anti-CD27 (LG.3A10; FITC), anti-CD8α (53-6.7; FITC), anti-TCRβ (H57-597; FITC), anti-CD4 (RM4-5; FITC), anti-Cxcr3 (CXCR3-173; RB744), anti-Nur77 (12.14; PerCP–eFluor 710), anti–IL-22 (1H8BWSR; PerCP–eFluor 710), anti-TCRVγ2 (UC3-10A6; PerCP–eFluor 710), anti-CCR6 (29-2 L17; allophycocyanin (APC); 1:100), anti-CD3ε (145-2C11; APC), anti–IFN-γ (XMG1.2; APC), anti-CD44 (IM7; Alexa Fluor 700; 1:100), anti-TCRβ (H57-597; APC–eFluor 780), anti-CD11b (M1/70; APC–eFluor 780), anti-TCRVγ6 [1C10-1F7; phycoerythrin (PE)], anti-RORγt (B2D; PE), anti-CD3ε (145-2C11; PE-CF594), anti–IL-17F (O79/289; PE-CF594), anti-RORγt (B2D; PE–eFluor 610), anti-CD27 (LG.3A10; PE-Cy7), anti-Tbet (4B10; PE-Cy7), anti-TCRγδ (GL3; PE-Cy7), anti-TCRγδ (GL3; BV421), anti-CD44 (IM7; V500), anti-CD45 (30F11; V500), anti-ICOS (C398.4A; BV605), anti-CD45.2 (104; BV650), anti-P2x7 (1F11/TSLPR; BV650), anti–IL-17A (TC11-18H10; BV-786), anti-Slamf7 (4G2; BV786), anti-CD4 (GK1.5; BUV-395), anti-CD45 (30F11; BUV-395), anti-CD49a (Ha31/8; BUV496), anti-Ly6A/E (E13 to E161.7; BUV563), anti-CD127 (SB/199; BUV-737; 1:100), and anti-CD3ε (145-2C11; BUV-737).

2) Human: αCD3 (OKT3; FITC; 1:50), anti-CD11b (M1/70; PerCP Cy5.5; 1:50), anti-RORγt (AFKJS-9; PE; 1:100), anti-Tbet (4B10; PE-Cy7; 1:100), anti-CD14 (MφP9; PE-CF594; 1:100), anti-CD19 (HIB19; APC; 1:20), anti-TCRVδ1 (TS8.2; APC, 1:20); anti-CD161 (HP-3G10; APC-Cy7; 1:20), anti-TCRγδ (11F2; BV421; 1:50), anti-CD4 (SK3; BV510; 1:50), anti-CD45 (HI30; BV605; 1:80), anti-CD127 (HIL-7R-M21; BV-650; 1:25), anti–human leukocyte antigen (HLA)–DR (L243; BV650; 1:100), anti-CD69 (FN50; BUV-395; 1:20), and anti–HLA-DR (L243; BUV737; 1:160).

### Gating strategies

Following FSC-A/SSC-A, FSC-H/FSC-W, SSC-H/SSC-W and Live/Dead gating, LN mouse γδ T cells were identified as CD8^−^CD4^−^CD19^−^TCRβ^−^CD11b^−^TCRγδ^+^ and thereafter γδT17 as RORγt^GFP^ or CD27^−^CD44^+^ or CD27^−^CD44^+^CCR6^+^ and γδT1 cells as Tbet^AmCyan^ or CD27^+^. Thymic γδ T cells were identified as CD8^−^CD4^−^TCRβ^−^TCRγδ^+^ and thereafter γδT17 as CD27^−^CD44^+^. LP mouse γδ T cells were identified as CD4^−^CD8^−^CD19^−^TCRβ^−^CD45^+^CD3^+^TCRγδ^+^ and thereafter γδT1 cells as RORγt^GFPneg^Tbet^AmCyan^ or RORγt*^−^*Tbet^+^ or CD45^+^IL-7Rα*^−^,* and γδT17 as RORγt^GFP^ or RORγt^+^ or CD45^int^IL-7Rα/CD127^hi^. ILC3 were identified as CD45^+^CD3*^−^*CD8^−^CD19^−^TCRβ*^−^*TCRγδ*^−^*CD127^+^. CD4 cells were identified as CD45^+^CD3^+^CD4^+^. For Tempo mice, FT Blue signal was detected in BV421 channel, and FT Red signal was detected in Texas Red channel. LP human γδ T cells were identified as CD19^−^CD11b^−^CD14^−^HLA-DR^−^CD4^−^CD45^+^CD3^+^TCRγδ*^+^* and subsequently CD69^+^RORγt^+^CD127^+/−^Tbet^+/*−*^ and CD69^−^RORγt*^−^*CD127^+/*−*^Tbet^+/*−*^ (donor 3) or CD69^+^CD161^+^RORγt^+/−^Tbet^+/*−*^ and CD69*^−^*CD161*^−^*RORγt^+/−^Tbet^+/−^ (donors 9, 6, and 8) (*62*)*.*

### Bulk RNA-seq

RORγt^GFP^ and/or Tbet^AmCyan^, RORγt^RFP^Tbet^F/F^ (Tbet-deficient), and neonatal (days 0 and 7) RORγt^GFP^ γδ T cells were sorted using a BD FACSAria Fusion III (BD Biosciences). Target populations were sorted directly in RNAprotect Cell Reagent (QIAGEN). Total RNA was extracted with the QIAGEN RNeasy Plus Micro Kit. The “Clontech SMARTer Ultra Low Input RNA v4” and the “Illumina Nextera XT” kits were used to generate mRNA-seq libraries, which were sequenced on an Illumina NextSeq 500 or 2000 instrument. RNA extraction, library preparation, and RNA-seq were performed at the Genomics Core Facility “KFB - Center of Excellence for Fluorescent Bioanalytics” (University of Regensburg, Regensburg, Germany; www.kfb-regensburg.de) (*62*).

### Bulk RNA-seq analysis

As reported in (*62*), all samples passed quality control with FastQC v0.11.2 (www.bioinformatics.babraham.ac.uk/projects/fastqc/). Reads were mapped to GRCm39 Ensembl release 109 (36318249) using kallisto v0.48.0 (27043002). Gene quantifications were imported into R using tximport v1.26.1 (26925227), and differential expression was calculated using a quasi-likelihood test with edgeR v3.40.2 (19910308). GSEA (16199517) was performed using fgsea v1.28.0 (https://doi.org/10.1101/060012) using weighted signed −log_10_ differential expression *P* values as test statistic. Single-sample GSEA (19847166) was performed using gene set variation analysis v1.46.0 (23323831). Visualizations were generated using ggplot2 v3.4.4 (*64*) and ComplexHeatmap v2.14.0 (27207943) using R version 4.2.2 (*65*).

### Single-cell RNA sequencing

A total of 30,000 human γδ T cells were sorted from siLP, and 28,000 were sorted from cLP. Before sorting, single-cell suspensions were resuspended in MACS buffer at 10^8^ cells/ml containing 1:100 biotinylated anti-CD19 (HIB19), anti-TCRαβ (IP26), anti-CD14 (HCD14), and anti-CD11c (3.9). The cells were incubated for 15 min at room temperature and subsequently incubated with EasySep RaphidSphere streptavidin beads (75 μl/ml) for 2.5 min and transferred to an EasySep magnet for 2.5 min. Remaining cells were collected and stained with anti-CD45, anti-TCRγδ, anti–HLA-DR, anti-CD14, αCD3, and anti-CD19; sorted as HLA-DR^−^CD19^−^CD14^−^CD45^+^CD3^+^TCRγδ^+^ using a BD FACSAria Fusion III in cold PBS containing bovine serum albumin (0.5%); and diluted to the desired concentration following the 10x Genomics guidelines [Chromium Next GEM Single Cell 5′ v2 (Dual Index) User Guide, CG000331 Rev. E]. For the mouse samples, cells were sorted from Cre^−^ and Cre^+^ RORγt^CRE^-cIAP1^F/F^-cIAP2^KO^ mice (*29*), and each genotype was differentially hashed with TotalSeq anti-mouse antibodies (C0304 and C0305; 1 μg per 10^6^ cells; BioLegend). Cre^−^ (WT) cells were further analyzed as described below, whereas data from Cre^+^ cells were irrelevant to this project. Cells were sorted as total γδ T cells. scRNA-seq libraries were prepared according to the manufacturer’s instructions using Chromium Next GEM Single Cell 5′ Kit v2 (PN-1000265, 10x Genomics), Chromium Next GEM Chip K Single Cell Kit (PN-1000287, 10x Genomics), and Dual Index Kit TT Set A (PN-1000215, 10x Genomics) with the Chromium Controller (PN-120223) or the Chromium Single Cell Controller (PN-120263). Library quality control and quantification were performed using KAPA Library Quantification Kit for Illumina Platforms (KK4824, Kapa Biosystems) and the 2100 Bioanalyzer equipped with a High Sensitivity DNA kit (5067-4626, Agilent). Libraries were sequenced on an Illumina NovaSeq 6000 instrument at the Flow Cytometry & Single Cell Core Facility at the University of Copenhagen, Denmark (*62*).

### scRNA-seq analysis

All human samples passed quality control with Fastqc v0.11.2. CellRanger v7.1.0 was used to align the reads to the GRCh38 version 2020-A reference genome, as well as for filtering cell and unique molecular identifier barcodes and quantifying gene expression per cell. Seurat v5.0.1 was used for further quality control and processing (*66*). Samples taken from the colon and the small intestine were analyzed separately but followed the same processing workflow. Cells with a mitochondrial count exceeding 4% were removed from both samples and cells expressing more than 5% of heat shock proteins (colon sample) and more than 2% (small intestine sample). To further remove low-quality cells and possible doublets, cells with less than 200 genes and cells with more than 4000 genes were discarded from both samples. Count matrices were normalized and scaled using Seurat’s SCTransform function. The first 15 principal components (colon sample) and the first 20 principal components (small intestine sample) were used for clustering as they captured over 70% of variance. *K*-nearest neighbor clustering was then applied to identify distinct cell clusters. Visualization of these clusters was done through UMAP graphs with a resolution of 0.1.

For the mouse dataset, samples were aligned to the GRCm39-2024-A reference genome using CellRanger v8.0.0. Seurat v5.1.0 was used for further preprocessing and analysis. Data from each time point were processed individually. HTODemux was used to demultiplex cells from each genotype (Cre^−^ and Cre^+^) and to remove doublets and nonhashed cells. For this manuscript, only data from Cre^−^ mice were included in the analysis. Cells with mitochondrial counts higher than 5% and those with low or high number of RNA molecules and genes were excluded. In addition, all ribosomal genes were removed. Afterward, preprocessed objects were merged (days 0, 7, 21, and 42). The data were log normalized, the effect of cell cycle was regressed out, and Harmony was used for integration. We found contamination from myeloid and other non–γδ T cells in our samples, and these were removed. Then, Vγ4 and Vγ6 cells were subsetted on the basis of the condition that cells express at least one transcript of *Trgv4* or *Trgv6*, respectively. After subsetting, the data were rescaled and reintegrated. Unsupervised clustering was run with Seurat’s default parameters.

Cluster identities were found running Seurat’s “FindAllMarkers” function. Expression of the selected genes per cluster was quantified through Seurat’s AverageExpression function and further illustrated using the DoHeatmap function. DEGs were identified using Seurat’s “FindMarkers” function. Only genes expressed in at least 25% of cells and with a log_2_ fold change (log_2_FC) of >1 and an adjusted *P* value of <0.05 (determined using a Wilcoxon rank sum test) were considered. In addition, coexpression analysis for human *RORC* was conducted computing the Pearson correlation coefficient and *P* value for each gene against the selected two genes. Multiple testing correction was applied using Bonferroni correction, considering genes with a corrected *P* value less than 0.05 as statistically significant. The top 35 genes exhibiting the most significant coexpression with *RORC* were visually presented in a bar chart. Subsequently, the percentage of cells in *RORC*^+^ cell populations expressing the genes (*MAF*, *CCR6*, *KLRB1*, *DPP4*, *RORA*, *IL23R*, *CXCR6*, *BLK*, *BHLHE40*, and *ZBTB16*) was calculated and visually represented (*62*).

### Statistics

Unless otherwise stated, all the data were statistically analyzed using GraphPad Prism v9 or v10. Statistical tests depended on the nature of the data and are indicated in the figure legend.

## References

[1] J. C. Ribot, A. deBarros, D. J. Pang, J. F. Neves, V. Peperzak, S. J. Roberts, M. Girardi, J. Borst, A. C. Hayday, D. J. Pennington, B. Silva-Santos, CD27 is a thymic determinant of the balance between interferon-γ- and interleukin 17-producing γδ T cell subsets. Nat. Immunol. 10, 427–436 (2009). 19270712 10.1038/ni.1717 PMC4167721

[2] J. D. Haas, S. Ravens, S. Duber, I. Sandrock, L. Oberdorfer, E. Kashani, V. Chennupati, L. Fohse, R. Naumann, S. Weiss, A. Krueger, R. Forster, I. Prinz, Development of interleukin-17-producing γδ T cells is restricted to a functional embryonic wave. Immunity 37, 48–59 (2012). 22770884 10.1016/j.immuni.2012.06.003

[3] N. Sumaria, C. L. Grandjean, B. Silva-Santos, D. J. Pennington, Strong TCRγδ signaling prohibits thymic development of IL-17A-secreting γδ T cells. Cell Rep. 19, 2469–2476 (2017). 28636936 10.1016/j.celrep.2017.05.071 PMC5489697

[4] N. Sumaria, S. Martin, D. J. Pennington, Constrained TCRγδ-associated Syk activity engages PI3K to facilitate thymic development of IL-17A-secreting γδ T cells. Sci. Signal. 14, eabc5884 (2021). 34285131 10.1126/scisignal.abc5884

[5] M. Munoz-Ruiz, J. C. Ribot, A. R. Grosso, N. Goncalves-Sousa, A. Pamplona, D. J. Pennington, J. R. Regueiro, E. Fernandez-Malave, B. Silva-Santos, TCR signal strength controls thymic differentiation of discrete proinflammatory γδ T cell subsets. Nat. Immunol. 17, 721–727 (2016). 27043412 10.1038/ni.3424 PMC4875770

[6] G. Turchinovich, A. C. Hayday, Skint-1 identifies a common molecular mechanism for the development of interferon-γ-secreting versus interleukin-17-secreting γδ T cells. Immunity 35, 59–68 (2011). 21737317 10.1016/j.immuni.2011.04.018

[7] C. E. Sutton, S. J. Lalor, C. M. Sweeney, C. F. Brereton, E. C. Lavelle, K. H. Mills, Interleukin-1 and IL-23 induce innate IL-17 production from γδ T cells, amplifying Th17 responses and autoimmunity. Immunity 31, 331–341 (2009). 19682929 10.1016/j.immuni.2009.08.001

[8] Y. Cai, X. Shen, C. Ding, C. Qi, K. Li, X. Li, V. R. Jala, H. G. Zhang, T. Wang, J. Zheng, J. Yan, Pivotal role of dermal IL-17-producing γδ T cells in skin inflammation. Immunity 35, 596–610 (2011). 21982596 10.1016/j.immuni.2011.08.001 PMC3205267

[9] V. Bekiaris, J. R. Sedy, M. G. Macauley, A. Rhode-Kurnow, C. F. Ware, The inhibitory receptor BTLA controls γδ T cell homeostasis and inflammatory responses. Immunity 39, 1082–1094 (2013). 24315996 10.1016/j.immuni.2013.10.017 PMC3909738

[10] M. Rei, N. Goncalves-Sousa, T. Lanca, R. G. Thompson, S. Mensurado, F. R. Balkwill, H. Kulbe, D. J. Pennington, B. Silva-Santos, Murine CD27^−^ Vγ6^+^ γδ T cells producing IL-17A promote ovarian cancer growth via mobilization of protumor small peritoneal macrophages. Proc. Natl. Acad. Sci. U.S.A. 111, E3562–E3570 (2014). 25114209 10.1073/pnas.1403424111 PMC4151711

[11] A. C. Kohlgruber, S. T. Gal-Oz, N. M. LaMarche, M. Shimazaki, D. Duquette, H. F. Koay, H. N. Nguyen, A. I. Mina, T. Paras, A. Tavakkoli, U. von Andrian, A. P. Uldrich, D. I. Godfrey, A. S. Banks, T. Shay, M. B. Brenner, L. Lynch, γδ T cells producing interleukin-17A regulate adipose regulatory T cell homeostasis and thermogenesis. Nat. Immunol. 19, 464–474 (2018). 29670241 10.1038/s41590-018-0094-2 PMC8299914

[12] M. Ribeiro, H. C. Brigas, M. Temido-Ferreira, P. A. Pousinha, T. Regen, C. Santa, J. E. Coelho, I. Marques-Morgado, C. A. Valente, S. Omenetti, B. Stockinger, A. Waisman, B. Manadas, L. V. Lopes, B. Silva-Santos, J. C. Ribot, Meningeal γδ T cell-derived IL-17 controls synaptic plasticity and short-term memory. Sci. Immunol. 4, eaay5199 (2019). 31604844 10.1126/sciimmunol.aay5199 PMC6894940

[13] K. Alves de Lima, J. Rustenhoven, S. Da Mesquita, M. Wall, A. F. Salvador, I. Smirnov, G. Martelossi Cebinelli, T. Mamuladze, W. Baker, Z. Papadopoulos, M. B. Lopes, W. S. Cao, X. S. Xie, J. Herz, J. Kipnis, Meningeal γδ T cells regulate anxiety-like behavior via IL-17a signaling in neurons. Nat. Immunol. 21, 1421–1429 (2020). 32929273 10.1038/s41590-020-0776-4 PMC8496952

[14] J. S. Heilig, S. Tonegawa, Diversity of murine γ genes and expression in fetal and adult T lymphocytes. Nature 322, 836–840 (1986). 2943999 10.1038/322836a0

[15] R. Agerholm, V. Bekiaris, Evolved to protect, designed to destroy: IL-17-producing γδ T cells in infection, inflammation, and cancer. Eur. J. Immunol. 51, 2164–2177 (2021). 34224140 10.1002/eji.202049119

[16] J. C. Ribot, N. Lopes, B. Silva-Santos, γδ T cells in tissue physiology and surveillance. Nat. Rev. Immunol. 21, 221–232 (2021). 33057185 10.1038/s41577-020-00452-4

[17] L. Tan, A. S. Fichtner, E. Bruni, I. Odak, I. Sandrock, A. Bubke, A. Borchers, C. Schultze-Florey, C. Koenecke, R. Forster, M. Jarek, C. von Kaisenberg, A. Schulz, X. Chu, B. Zhang, Y. Li, U. Panzer, C. F. Krebs, S. Ravens, I. Prinz, A fetal wave of human type 3 effector γδ cells with restricted TCR diversity persists into adulthood. Sci. Immunol. 6, (2021). 10.1126/sciimmunol.abf0125 33893173

[18] P. L. Ryan, N. Sumaria, C. J. Holland, C. M. Bradford, N. Izotova, C. L. Grandjean, A. S. Jawad, L. A. Bergmeier, D. J. Pennington, Heterogeneous yet stable Vdelta2^(+)^ T-cell profiles define distinct cytotoxic effector potentials in healthy human individuals. Proc. Natl. Acad. Sci. U.S.A. 113, 14378–14383 (2016). 27911793 10.1073/pnas.1611098113 PMC5167212

[19] Y. Wu, F. Kyle-Cezar, R. T. Woolf, C. Naceur-Lombardelli, J. Owen, D. Biswas, A. Lorenc, P. Vantourout, P. Gazinska, A. Grigoriadis, A. Tutt, A. Hayday, An innate-like Vδ1^+^ γδ T cell compartment in the human breast is associated with remission in triple-negative breast cancer. Sci. Transl. Med. 11, (2019). 10.1126/scitranslmed.aax9364 PMC6877350 31597756

[20] P. Wu, D. Wu, C. Ni, J. Ye, W. Chen, G. Hu, Z. Wang, C. Wang, Z. Zhang, W. Xia, Z. Chen, K. Wang, T. Zhang, J. Xu, Y. Han, T. Zhang, X. Wu, J. Wang, W. Gong, S. Zheng, F. Qiu, J. Yan, J. Huang, γδT17 cells promote the accumulation and expansion of myeloid-derived suppressor cells in human colorectal cancer. Immunity 40, 785–800 (2014). 24816404 10.1016/j.immuni.2014.03.013 PMC4716654

[21] U. Laggner, P. Di Meglio, G. K. Perera, C. Hundhausen, K. E. Lacy, N. Ali, C. H. Smith, A. C. Hayday, B. J. Nickoloff, F. O. Nestle, Identification of a novel proinflammatory human skin-homing Vγ9Vδ2 T cell subset with a potential role in psoriasis. J. Immunol. 187, 2783–2793 (2011). 21813772 10.4049/jimmunol.1100804 PMC3187621

[22] L. Schirmer, V. Rothhammer, B. Hemmer, T. Korn, Enriched CD161^high^ CCR6^+^ γδ T cells in the cerebrospinal fluid of patients with multiple sclerosis. JAMA Neurol. 70, 345–351 (2013). 23599932 10.1001/2013.jamaneurol.409

[23] K. Venken, P. Jacques, C. Mortier, M. E. Labadia, T. Decruy, J. Coudenys, K. Hoyt, A. L. Wayne, R. Hughes, M. Turner, S. Van Gassen, L. Martens, D. Smith, C. Harcken, J. Wahle, C. T. Wang, E. Verheugen, N. Schryvers, G. Varkas, H. Cypers, R. Wittoek, Y. Piette, L. Gyselbrecht, S. Van Calenbergh, F. Van den Bosch, Y. Saeys, G. Nabozny, D. Elewaut, RORγt inhibition selectively targets IL-17 producing iNKT and γδ-T cells enriched in Spondyloarthritis patients. Nat. Commun. 10, 9 (2019). 30602780 10.1038/s41467-018-07911-6 PMC6315029

[24] D. Fenoglio, A. Poggi, S. Catellani, F. Battaglia, A. Ferrera, M. Setti, G. Murdaca, M. R. Zocchi, Vδ1 T lymphocytes producing IFN-γ and IL-17 are expanded in HIV-1-infected patients and respond to *Candida albicans*. Blood 113, 6611–6618 (2009). 19395673 10.1182/blood-2009-01-198028

[25] G. Sanchez Sanchez, M. Papadopoulou, A. Azouz, Y. Tafesse, A. Mishra, J. K. Y. Chan, Y. Fan, I. Verdebout, S. Porco, F. Libert, F. Ginhoux, B. Vandekerckhove, S. Goriely, D. Vermijlen, Identification of distinct functional thymic programming of fetal and pediatric human γδ thymocytes via single-cell analysis. Nat. Commun. 13, 5842 (2022). 36195611 10.1038/s41467-022-33488-2 PMC9532436

[26] B. A. Mangan, M. R. Dunne, V. P. O’Reilly, P. J. Dunne, M. A. Exley, D. O’Shea, E. Scotet, A. E. Hogan, D. G. Doherty, Cutting edge: CD1d restriction and Th1/Th2/Th17 cytokine secretion by human Vδ3 T cells. J. Immunol. 191, 30–34 (2013). 23740951 10.4049/jimmunol.1300121 PMC3721026

[27] N. Torow, M. W. Hornef, The neonatal window of opportunity: Setting the stage for life-long host-microbial interaction and immune homeostasis. J. Immunol. 198, 557–563 (2017). 28069750 10.4049/jimmunol.1601253

[28] D. Kadekar, R. Agerholm, J. Rizk, H. A. Neubauer, T. Suske, B. Maurer, M. T. Vinals, E. M. Comelli, A. Taibi, R. Moriggl, V. Bekiaris, The neonatal microenvironment programs innate γδ T cells through the transcription factor STAT5. J. Clin. Invest. 130, 2496–2508 (2020). 32281944 10.1172/JCI131241 PMC7190909

[29] J. Rizk, U. M. Morbe, R. Agerholm, M. V. Baglioni, E. Catafal Tardos, M. G. F. Fares da Silva, I. Ulmert, D. Kadekar, M. T. Vinals, V. Bekiaris, The cIAP ubiquitin ligases sustain type 3 γδ T cells and ILC during aging to promote barrier immunity. J. Exp. Med. 220, e20221534 (2023). 37440178 10.1084/jem.20221534 PMC10345214

[30] R. Di Marco Barros, N. A. Roberts, R. J. Dart, P. Vantourout, A. Jandke, O. Nussbaumer, L. Deban, S. Cipolat, R. Hart, M. L. Iannitto, A. Laing, B. Spencer-Dene, P. East, D. Gibbons, P. M. Irving, P. Pereira, U. Steinhoff, A. Hayday, Epithelia use butyrophilin-like molecules to shape organ-specific γδ T cell compartments. Cell 167, 203–218.e17 (2016). 27641500 10.1016/j.cell.2016.08.030 PMC5037318

[31] P. H. Papotto, B. Yilmaz, G. Pimenta, S. Mensurado, C. Cunha, G. J. Fiala, D. Gomes da Costa, N. Goncalves-Sousa, B. H. K. Chan, B. Blankenhaus, R. G. Domingues, T. Carvalho, M. R. Hepworth, A. J. Macpherson, J. E. Allen, B. Silva-Santos, Maternal γδ T cells shape offspring pulmonary type 2 immunity in a microbiota-dependent manner. Cell Rep. 42, 112074 (2023). 36787741 10.1016/j.celrep.2023.112074 PMC7615642

[32] N. A. Spidale, N. Malhotra, M. Frascoli, K. Sylvia, B. Miu, C. Freeman, B. D. Stadinski, E. Huseby, J. Kang, Neonatal-derived IL-17 producing dermal γδ T cells are required to prevent spontaneous atopic dermatitis. eLife 9, (2020). 10.7554/eLife.51188 PMC7025821 32065580

[33] E. Moens, M. Brouwer, T. Dimova, M. Goldman, F. Willems, D. Vermijlen, IL-23R and TCR signaling drives the generation of neonatal Vγ9Vδ2 T cells expressing high levels of cytotoxic mediators and producing IFN-γand IL-17. J. Leukoc. Biol. 89, 743–752 (2011). 21330350 10.1189/jlb.0910501

[34] M. L. Michel, D. J. Pang, S. F. Haque, A. J. Potocnik, D. J. Pennington, A. C. Hayday, Interleukin 7 (IL-7) selectively promotes mouse and human IL-17-producing γδ cells. Proc. Natl. Acad. Sci. U.S.A. 109, 17549–17554 (2012). 23047700 10.1073/pnas.1204327109 PMC3491488

[35] S. Ravens, A. S. Fichtner, M. Willers, D. Torkornoo, S. Pirr, J. Schoning, M. Deseke, I. Sandrock, A. Bubke, A. Wilharm, D. Dodoo, B. Egyir, K. L. Flanagan, L. Steinbruck, P. Dickinson, P. Ghazal, B. Adu, D. Viemann, I. Prinz, Microbial exposure drives polyclonal expansion of innate γδ T cells immediately after birth. Proc. Natl. Acad. Sci. U.S.A. 117, 18649–18660 (2020). 32690687 10.1073/pnas.1922588117 PMC7414158

[36] M. Papadopoulou, T. Dimova, M. Shey, L. Briel, H. Veldtsman, N. Khomba, H. Africa, M. Steyn, W. A. Hanekom, T. J. Scriba, E. Nemes, D. Vermijlen, Fetal public Vγ9Vδ2 T cells expand and gain potent cytotoxic functions early after birth. Proc. Natl. Acad. Sci. U.S.A. 117, 18638–18648 (2020). 32665435 10.1073/pnas.1922595117 PMC7414170

[37] H. R. Conti, A. C. Peterson, L. Brane, A. R. Huppler, N. Hernandez-Santos, N. Whibley, A. V. Garg, M. R. Simpson-Abelson, G. A. Gibson, A. J. Mamo, L. C. Osborne, S. Bishu, N. Ghilardi, U. Siebenlist, S. C. Watkins, D. Artis, M. J. McGeachy, S. L. Gaffen, Oral-resident natural Th17 cells and γδ T cells control opportunistic *Candida albicans* infections. J. Exp. Med. 211, 2075–2084 (2014). 25200028 10.1084/jem.20130877 PMC4172215

[38] R. Wiesheu, S. C. Edwards, A. Hedley, H. Hall, M. Tosolini, M. G. F. Fares da Silva, N. Sumaria, S. M. Castenmiller, L. Wardak, Y. Optaczy, A. Lynn, D. G. Hill, A. J. Hayes, J. Hay, A. Kilbey, R. Shaw, D. Whyte, P. J. Walsh, A. M. Michie, G. J. Graham, A. Manoharan, C. Halsey, K. Blyth, M. C. Wolkers, C. Miller, D. J. Pennington, G. W. Jones, J. J. Fournie, V. Bekiaris, S. B. Coffelt, IL-27 maintains cytotoxic Ly6C^+^ γδ T cells that arise from immature precursors. EMBO J. 43, 2878–2907 (2024). 38816652 10.1038/s44318-024-00133-1 PMC11251046

[39] L. Drujont, A. Lemoine, A. Moreau, G. Bienvenu, M. Lancien, T. Cens, F. Guillot, G. Beriou, L. Bouchet-Delbos, H. J. Fehling, E. Chiffoleau, A. B. Nicot, P. Charnet, J. C. Martin, R. Josien, M. C. Cuturi, C. Louvet, RORγt^+^ cells selectively express redundant cation channels linked to the Golgi apparatus. Sci. Rep. 6, 23682 (2016). 27009467 10.1038/srep23682 PMC4806298

[40] Z. Al Nabhani, G. Eberl, Imprinting of the immune system by the microbiota early in life. Mucosal Immunol. 13, 183–189 (2020). 31988466 10.1038/s41385-020-0257-y

[41] C. Vonarbourg, A. Mortha, V. L. Bui, P. P. Hernandez, E. A. Kiss, T. Hoyler, M. Flach, B. Bengsch, R. Thimme, C. Holscher, M. Honig, U. Pannicke, K. Schwarz, C. F. Ware, D. Finke, A. Diefenbach, Regulated expression of nuclear receptor RORγt confers distinct functional fates to NK cell receptor-expressing RORγt^+^ innate lymphocytes. Immunity 33, 736–751 (2010). 21093318 10.1016/j.immuni.2010.10.017 PMC3042726

[42] M. E. Parker, A. Barrera, J. D. Wheaton, M. K. Zuberbuehler, D. S. J. Allan, J. R. Carlyle, T. E. Reddy, M. Ciofani, c-Maf regulates the plasticity of group 3 innate lymphoid cells by restraining the type 1 program. J. Exp. Med. 217, e20191030 (2020). 31570496 10.1084/jem.20191030 PMC7037249

[43] M. E. Parker, N. U. Mehta, T. C. Liao, W. H. Tomaszewski, S. A. Snyder, J. Busch, M. Ciofani, Restriction of innate Tγδ17 cell plasticity by an AP-1 regulatory axis. Nat. Immunol. 26, 1299–1314 (2025). 40579554 10.1038/s41590-025-02206-7 PMC12335200

[44] M. Wencker, G. Turchinovich, R. Di Marco Barros, L. Deban, A. Jandke, A. Cope, A. C. Hayday, Innate-like T cells straddle innate and adaptive immunity by altering antigen-receptor responsiveness. Nat. Immunol. 15, 80–87 (2014). 24241693 10.1038/ni.2773 PMC6485477

[45] D. Bending, J. Zikherman, Nr4a nuclear receptors: Markers and modulators of antigen receptor signaling. Curr. Opin. Immunol. 81, 102285 (2023). 36764055 10.1016/j.coi.2023.102285

[46] V. J. Brock, I. M. A. Wolf, M. Er-Lukowiak, N. Lory, T. Stahler, L. M. Woelk, H. W. Mittrucker, C. E. Muller, F. Koch-Nolte, B. Rissiek, R. Werner, A. H. Guse, B. P. Diercks, P2X4 and P2X7 are essential players in basal T cell activity and Ca^2+^ signaling milliseconds after T cell activation. Sci. Adv. 8, eabl9770 (2022). 35119925 10.1126/sciadv.abl9770 PMC8816335

[47] T. A. E. Elliot, E. K. Jennings, D. A. J. Lecky, S. Rouvray, G. M. Mackie, L. Scarfe, L. Sheriff, M. Ono, K. M. Maslowski, D. Bending, Nur77-Tempo mice reveal T cell steady state antigen recognition. Discov. Immunol. 1, kyac009 (2022). 36704407 10.1093/discim/kyac009 PMC7614040

[48] M. Frascoli, E. Ferraj, B. Miu, J. Malin, N. A. Spidale, J. Cowan, S. C. Shissler, R. Brink, Y. Xu, J. G. Cyster, A. Bhandoola, J. Kang, A. Reboldi, Skin γδ T cell inflammatory responses are hardwired in the thymus by oxysterol sensing via GPR183 and calibrated by dietary cholesterol. Immunity 56, 562–575.e6 (2023). 36842431 10.1016/j.immuni.2023.01.025 PMC10306310

[49] N. Torow, B. J. Marsland, M. W. Hornef, E. S. Gollwitzer, Neonatal mucosal immunology. Mucosal Immunol. 10, 5–17 (2017). 27649929 10.1038/mi.2016.81

[50] P. Henneke, K. Kierdorf, L. J. Hall, M. Sperandio, M. Hornef, Perinatal development of innate immune topology. eLife 10, e67793 (2021). 34032570 10.7554/eLife.67793 PMC8149122

[51] B. D. Rudd, Neonatal T cells: A reinterpretation. Annu. Rev. Immunol. 38, 229–247 (2020). 31928469 10.1146/annurev-immunol-091319-083608 PMC7369171

[52] S. Vergani, K. G. Muleta, C. Da Silva, A. Doyle, T. A. Kristiansen, S. Sodini, N. Krausse, G. Montano, K. Kotarsky, J. Nakawesi, H. Akerstrand, S. Vanhee, S. L. Gupta, D. Bryder, W. W. Agace, K. Lahl, J. Yuan, A self-sustaining layer of early-life-origin B cells drives steady-state IgA responses in the adult gut. Immunity 55, 1829–1842.e6 (2022). 36115337 10.1016/j.immuni.2022.08.018

[53] V. Lazarevic, L. H. Glimcher, G. M. Lord, T-bet: A bridge between innate and adaptive immunity. Nat. Rev. Immunol. 13, 777–789 (2013). 24113868 10.1038/nri3536 PMC6290922

[54] C. S. Klose, E. A. Kiss, V. Schwierzeck, K. Ebert, T. Hoyler, Y. d’Hargues, N. Goppert, A. L. Croxford, A. Waisman, Y. Tanriver, A. Diefenbach, A T-bet gradient controls the fate and function of CCR6^−^RORγt^+^ innate lymphoid cells. Nature 494, 261–265 (2013). 23334414 10.1038/nature11813

[55] T. Krausgruber, C. Schiering, K. Adelmann, O. J. Harrison, A. Chomka, C. Pearson, P. P. Ahern, M. Shale, M. Oukka, F. Powrie, T-bet is a key modulator of IL-23-driven pathogenic CD4^+^ T cell responses in the intestine. Nat. Commun. 7, 11627 (2016). 27193261 10.1038/ncomms11627 PMC4874038

[56] R. Duhen, S. Glatigny, C. A. Arbelaez, T. C. Blair, M. Oukka, E. Bettelli, Cutting edge: The pathogenicity of IFN-γ-producing Th17 cells is independent of T-bet. J. Immunol. 190, 4478–4482 (2013). 23543757 10.4049/jimmunol.1203172 PMC3633668

[57] V. Brucklacher-Waldert, C. Ferreira, S. Innocentin, S. Kamdar, D. R. Withers, M. C. Kullberg, M. Veldhoen, Tbet or continued RORγt expression is not required for Th17-associated immunopathology. J. Immunol. 196, 4893–4904 (2016). 27183623 10.4049/jimmunol.1600137 PMC4891569

[58] L. Tan, I. Sandrock, I. Odak, Y. Aizenbud, A. Wilharm, J. Barros-Martins, Y. Tabib, A. Borchers, T. Amado, L. Gangoda, M. J. Herold, M. Schmidt-Supprian, J. Kisielow, B. Silva-Santos, C. Koenecke, A. H. Hovav, C. Krebs, I. Prinz, S. Ravens, Single-cell transcriptomics identifies the adaptation of Scart1^+^ Vγ6^+^ T cells to skin residency as activated effector cells. Cell Rep. 27, 3657–3671.e4 (2019). 31216482 10.1016/j.celrep.2019.05.064

[59] J. L. Riley, M. Mao, S. Kobayashi, M. Biery, J. Burchard, G. Cavet, B. P. Gregson, C. H. June, P. S. Linsley, Modulation of TCR-induced transcriptional profiles by ligation of CD28, ICOS, and CTLA-4 receptors. Proc. Natl. Acad. Sci. U.S.A. 99, 11790–11795 (2002). 12195015 10.1073/pnas.162359999 PMC129347

[60] S. Meraviglia, E. Lo Presti, M. Tosolini, C. La Mendola, V. Orlando, M. Todaro, V. Catalano, G. Stassi, G. Cicero, S. Vieni, J. J. Fournie, F. Dieli, Distinctive features of tumor-infiltrating γδ T lymphocytes in human colorectal cancer. Onco Targets Ther 6, e1347742 (2017). 10.1080/2162402X.2017.1347742 PMC5665062 29123962

[61] M. Lochner, L. Peduto, M. Cherrier, S. Sawa, F. Langa, R. Varona, D. Riethmacher, M. Si-Tahar, J. P. Di Santo, G. Eberl, In vivo equilibrium of proinflammatory IL-17^+^ and regulatory IL-10^+^ Foxp3^+^ RORγt^+^ T cells. J. Exp. Med. 205, 1381–1393 (2008). 18504307 10.1084/jem.20080034 PMC2413035

[62] M. Fares da Silva, “Transcriptional, phenotypical and functional characterization of embryonic, neonatal and neuropathogenic γδ T cells,” thesis, Technical University of Denmark (2025).

[63] F. A. Schonherr, F. Sparber, F. R. Kirchner, E. Guiducci, K. Trautwein-Weidner, A. Gladiator, N. Sertour, U. Hetzel, G. T. T. Le, N. Pavelka, C. d’Enfert, M. E. Bougnoux, C. F. Corti, S. LeibundGut-Landmann, The intraspecies diversity of *C. albicans* triggers qualitatively and temporally distinct host responses that determine the balance between commensalism and pathogenicity. Mucosal Immunol. 10, 1335–1350 (2017). 28176789 10.1038/mi.2017.2

[64] H. Wickham, Ggplot2: Elegant graphics for data analysis (Springer, 2009), pp. viii, 212 p.

[65] R. C. Team, R: A language and environment for statistical computing. R Foundation for Statistical Computing, Vienna, Austria, The R Foundation (2021); www.R-project.org/.

[66] T. Stuart, A. Butler, P. Hoffman, C. Hafemeister, E. Papalexi, W. M. Mauck III, Y. Hao, M. Stoeckius, P. Smibert, R. Satija, Comprehensive integration of single-cell data. Cell 177, 1888–1902.e21 (2019). 31178118 10.1016/j.cell.2019.05.031 PMC6687398

