# Supplementary material for: A temporal differentiation program after birth delineates γδ T cell immunity
Source: Sci Adv. 2026 Aug 5;12(32):eaee6790. doi: 10.1126/sciadv.aee6790 (PMC13440409; doi:10.1126/sciadv.aee6790)
Supplement: Supplementary file 1 [file sciadv.aee6790_sm.pdf]

Science Advances
AAAS

# Supplementary Materials for

## A temporal differentiation program after birth delineates γδ T cell immunity

Maria V. Baglioni *et al.*

Corresponding author: Vasileios Bekiaris, vasbek@dtu.dk

*Sci. Adv.* **12**, eaee6790 (2026)
DOI: 10.1126/sciadv.aee6790

**The PDF file includes:**

Figs. S1 to S9
Legends for tables S1 to S4
Legend for data S1

**Other Supplementary Material for this manuscript includes the following:**

Tables S1 to S4
Data S1

## Supplementary Figures and Captions

# Figure S1

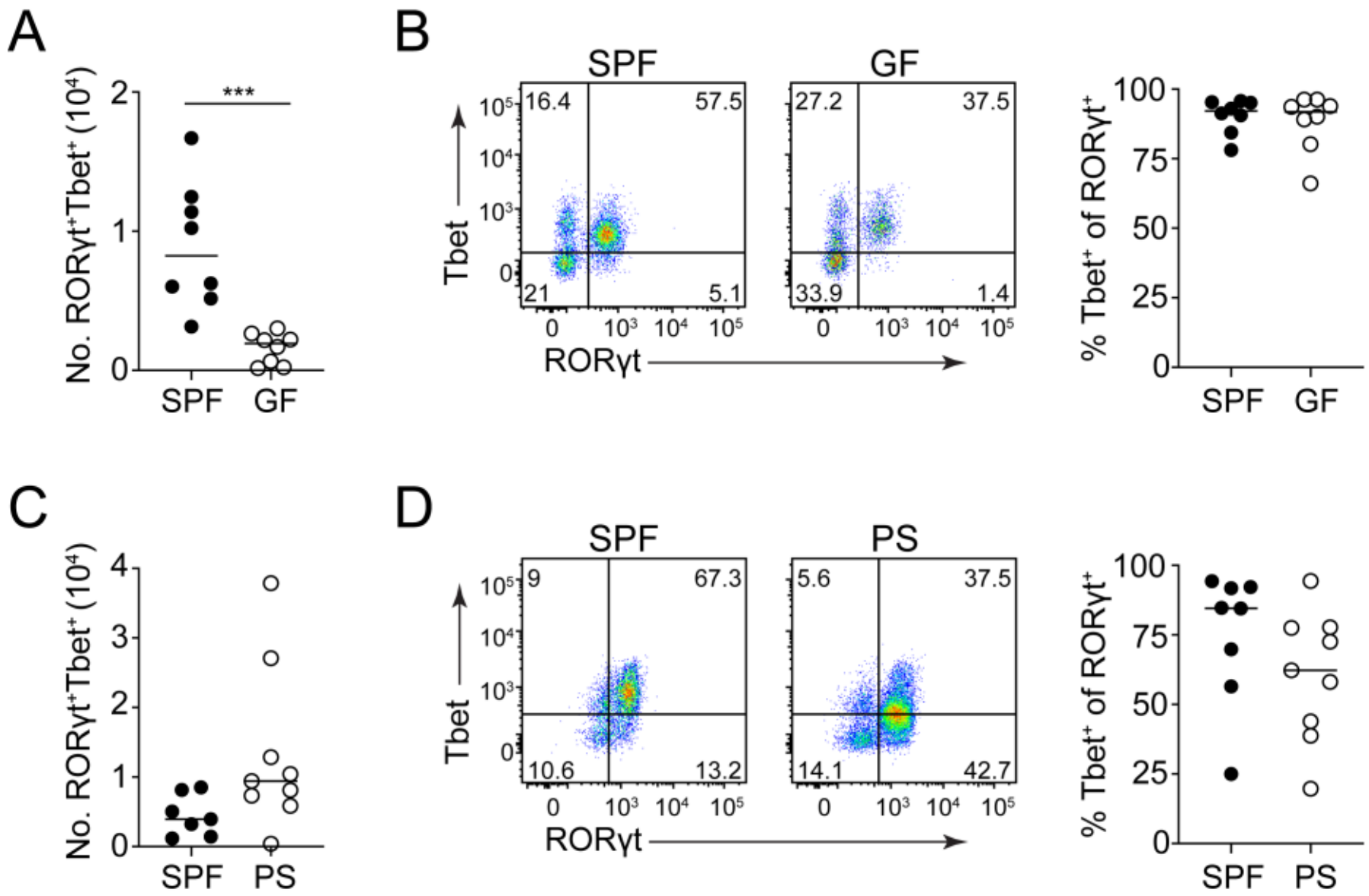


**Figure S1. Expression of Tbet in neonatal γδT17 cells is independent of the microbiota.** (**A**, **C**) Number of RORγt$^{+}$Tbet$^{+}$ γδ T cells in the siLP of specific pathogen free (SPF) and germ free (GF) mice (**A**) or mice bought from a local pet store (PS) (**C**). (**B**, **D**) Representative flow cytometric analysis (dot plots) of RORγt and Tbet expression in siLP γδ T cells and quantification (graphs) of Tbet expression within RORγt$^{+}$ γδ T cells in SPF and GF (**B**) or SPF and PS (**D**) mice; data from 3 (**A**, **B**) or 2 (**C**, **D**) experiments with 8 mice (**A**, **B**) and 8 or 9 mice (**C**, **D**). In all graphs each symbol represents a mouse and line the median; ***, $P < 0.001$ using the Mann-Whitney test.

# Figure S2

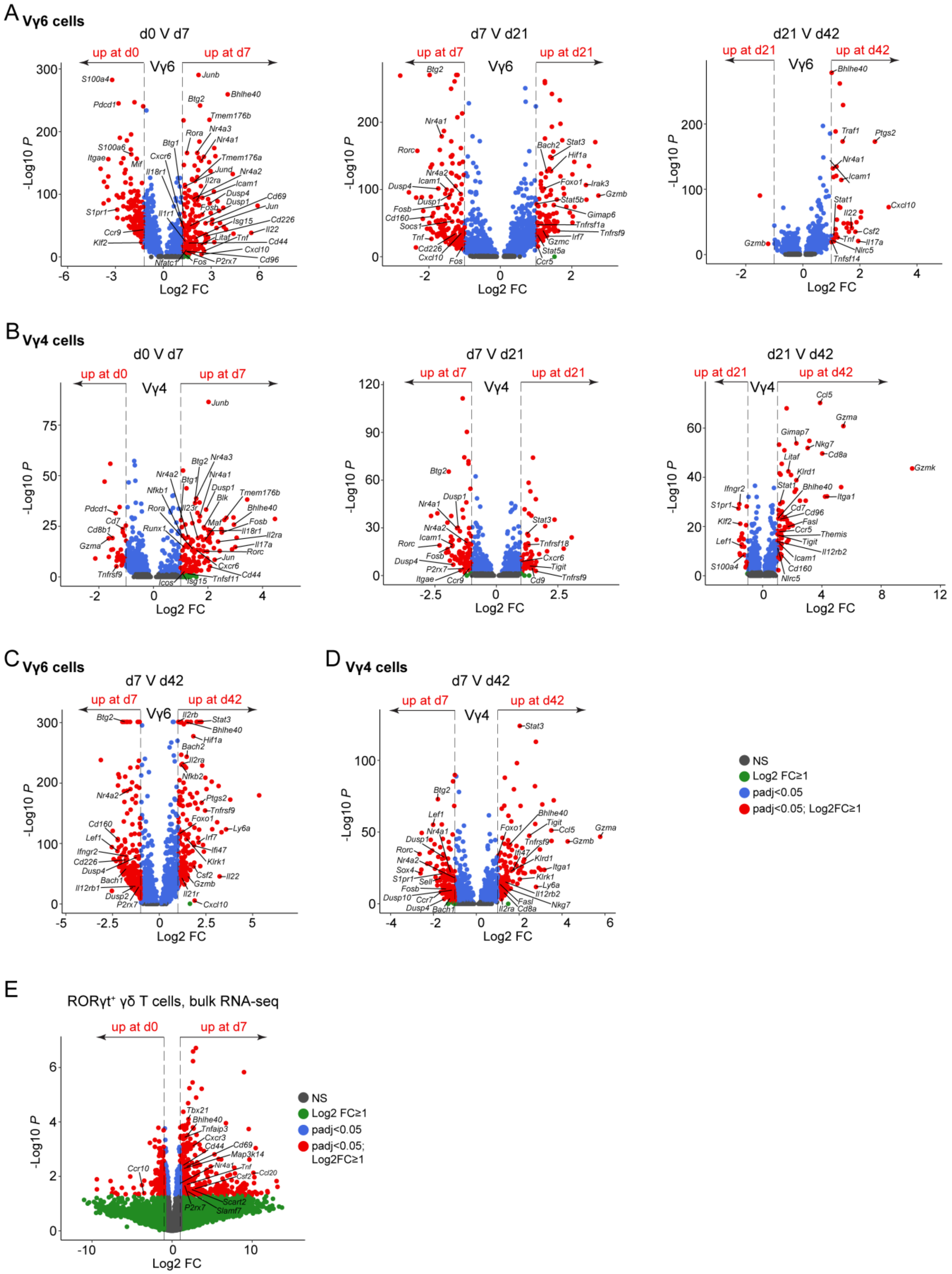
A
Vγ6 cells
d0 V d7
up at d0
up at d7
Vγ6
-Log10 P
Log2 FC
S100a4
Pdcd1
S100a6
Itgae
Mif
Il18r1
Cxcr6
Btg1
Rora
Btg2
Junb
Bhlhe40
Tmem176b
Nr4a3
Nr4a1
Tmem176a
Nr4a2
Il2ra
Jund
Icam1
Dusp4
Dusp1
Fosb
Cd69
Jun
Isg15
Cd226
Il22
Litaf
Tnf
Cd44
Cxcl10
Cd96
P2rx7
Fos
Nfatc1
Il1r1
S1pr1
Ccr9
Klf2
d7 V d21
up at d7
up at d21
Btg2
Nr4a1
Rorc
Nr4a2
Icam1
Dusp4
Dusp1
Fosb
Cd160
Socs1
Tnf
Cd226
Cxcl10
Fos
Stat3
Bach2
Hif1a
Foxo1
Irak3
Gzmb
Stat5b
Gimap6
Tnfrsf1a
Tnfrsf9
Irf7
Gzmc
Stat5a
Ccr5
d21 V d42
up at d21
up at d42
Bhlhe40
Traf1
Ptgs2
Nr4a1
Icam1
Stat1
Cxcl10
Il22
Csf2
Tnf
Il17a
Nlrc5
Tnfsf14
Gzmb
B
Vγ4 cells
d0 V d7
up at d0
up at d7
Vγ4
Junb
Btg2
Nr4a3
Nr4a2
Nr4a1
Btg1
Nfkb1
Dusp1
Blk
Tmem176b
Rora
Il23r
Bhlhe40
Maf
Fosb
Runx1
Il18r1
Il2ra
Il17a
Rorc
Jun
Cxcr6
Cd44
Tnfsf11
Isg15
Icos
Pdcd1
Cd7
Cd8b1
Gzma
Tnfrsf9
d7 V d21
up at d7
up at d21
Btg2
Dusp1
Nr4a1
Nr4a2
Icam1
Rorc
Fosb
Dusp4
P2rx7
Itgae
Ccr9
Stat3
Tnfrsf18
Cxcr6
Tigit
Tnfrsf9
Cd9
d21 V d42
up at d21
up at d42
Ccl5
Gzma
Gimap7
Nkg7
Cd8a
Litaf
Gzmk
Klrd1
Stat1
Bhlhe40
Itga1
Ifngr2
S1pr1
Cd7
Cd96
Klf2
Fasl
Ccr5
Themis
Lef1
Tigit
Il12rb2
Icam1
S100a4
Cd160
Nlrc5
C
Vγ6 cells
d7 V d42
up at d7
up at d42
Vγ6
Btg2
Il2rb
Stat3
Bhlhe40
Hif1a
Bach2
Il2ra
Nfkb2
Nr4a2
Ptgs2
Tnfrsf9
Foxo1
Ly6a
Cd160
Lef1
Irf7
Ifngr2
Ifi47
Cd226
Klrk1
Dusp4
Csf2
Il22
Bach1
Gzmb
Il12rb1
Il21r
Dusp2
P2rx7
Cxcl10
D
Vγ4 cells
d7 V d42
up at d7
up at d42
Vγ4
Stat3
Btg2
Lef1
Nr4a1
Dusp1
Rorc
Nr4a2
Sox4
S1pr1
Sell
Fosb
Dusp10
Ccr7
Dusp4
Bach1
Bhlhe40
Tigit
Foxo1
Ccl5
Gzma
Gzmb
Tnfrsf9
Ifi47
Klrd1
Itga1
Klrk1
Ly6a
Il12rb2
Fasl
Nkg7
Il2ra
Cd8a
NS
Log2 FC≥1
padj<0.05
padj<0.05; Log2FC≥1
E
RORγt⁺ γδ T cells, bulk RNA-seq
up at d0
up at d7
Tbx21
Bhlhe40
Tnfaip3
Cxcr3
Cd44
Cd69
Map3k14
Ccr10
Nr4a1
Tnf
Csf2
Ccl20
Scart2
P2rx7
Slamf7
NS
Log2 FC≥1
padj<0.05
padj<0.05; Log2FC≥1

**Figure S2. Differentially expressed genes in intestinal γδ T cells during neonatal life.**

(**A-D**) Volcano plots showing DEGs between the indicated time points of Vγ6 (**A**, **C**) or Vγ4 (**B**, **D**) γδ T cells. (**E**) Volcano plot indicating DEGs between d0 and d7 RORγt$^+$ γδ T cells (pooled siLP and cLP). (**A-E**) Each dot represents a gene; NS: not significant; selected genes highly expressed in each timepoint are labeled on the plot. In (**A-D**) γδ T cells were sorted from day 0 (d0; 10 mice; 1832 cells), d7 (9 mice; 3294 cells), d21 (3 mice; 4260 cells) and d42 (2 mice; 5915) siLP. In (**E**) data from 3 biological repeats, each consisting of a pool of 4 or 8 mice. P values were calculated using the Wilcoxon Rank Sum test (**A-D**) and a quasi-likelihood ratio test with the R package edgeR (**E**).

Figure S3

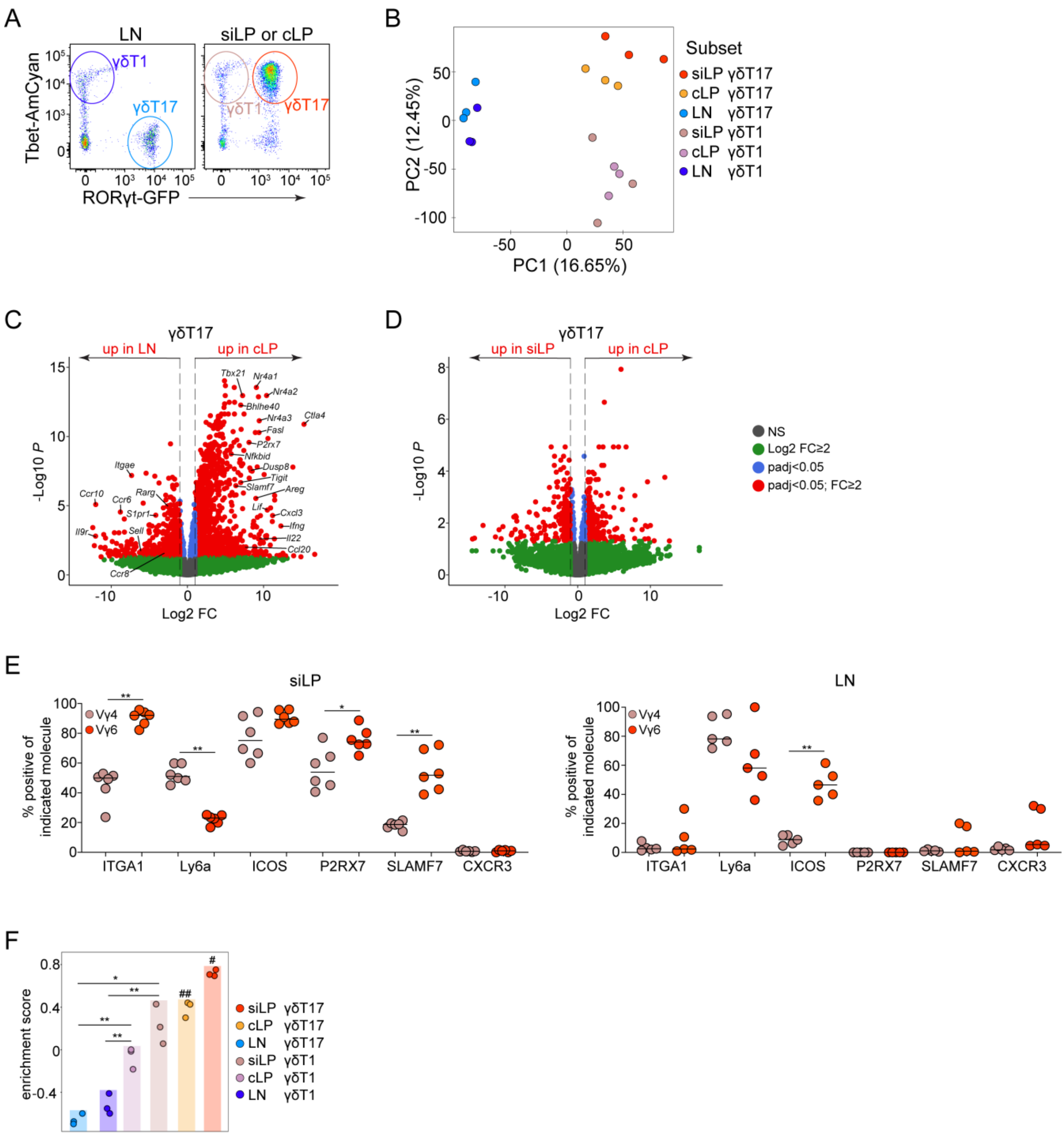


**Figure S3. Transcriptional and phenotypical analysis of γδ T cells from the LN and gut.**

(**A**) siLP or cLP and LN γδ T cells were sorted from RORγt$^{GFP}$Tbet$^{AmCyan}$ based on the GFP or AmCyan signal as shown. (**B**) PCA plot of the indicated γδ T cell populations following RNA-seq. (**C-D**) Volcano plots indicating DEGs between cLP and LN (**C**) or cLP and siLP (**D**) γδT17 cells; each dot represents a gene; NS: not significant; selected genes highly expressed in cLP, siLP or LN are labeled on the plot. (**E**) Quantification of expression of the indicated markers in Vγ4$^{+}$ or Vγ6$^{+}$ RORγt$^{+}$ γδ T cells either in siLP or LN; data from 2 experiments with 5 or 6 mice. Each

symbol represents a mouse and line the median. **(F)** Scatter plot showing the enrichment score of the intestinal γδT17 gene signature (gene list in **Fig. 3D**) for the indicated γδ T cells populations. In (**A-D**, **F**) data from 3 biological repeats, each consisting of a pool of 4-6 mice. P values were calculated using a quasi-likelihood ratio test with the R package edgeR (**C-D, F**) or the Mann-Whitney test **(E)**; *, $P < 0.05$; **, $P < 0.01$; $^{\#}$, significant against all comparisons; $^{\#\#}$, significant against all comparisons but siLP γδT1.

Figure S4

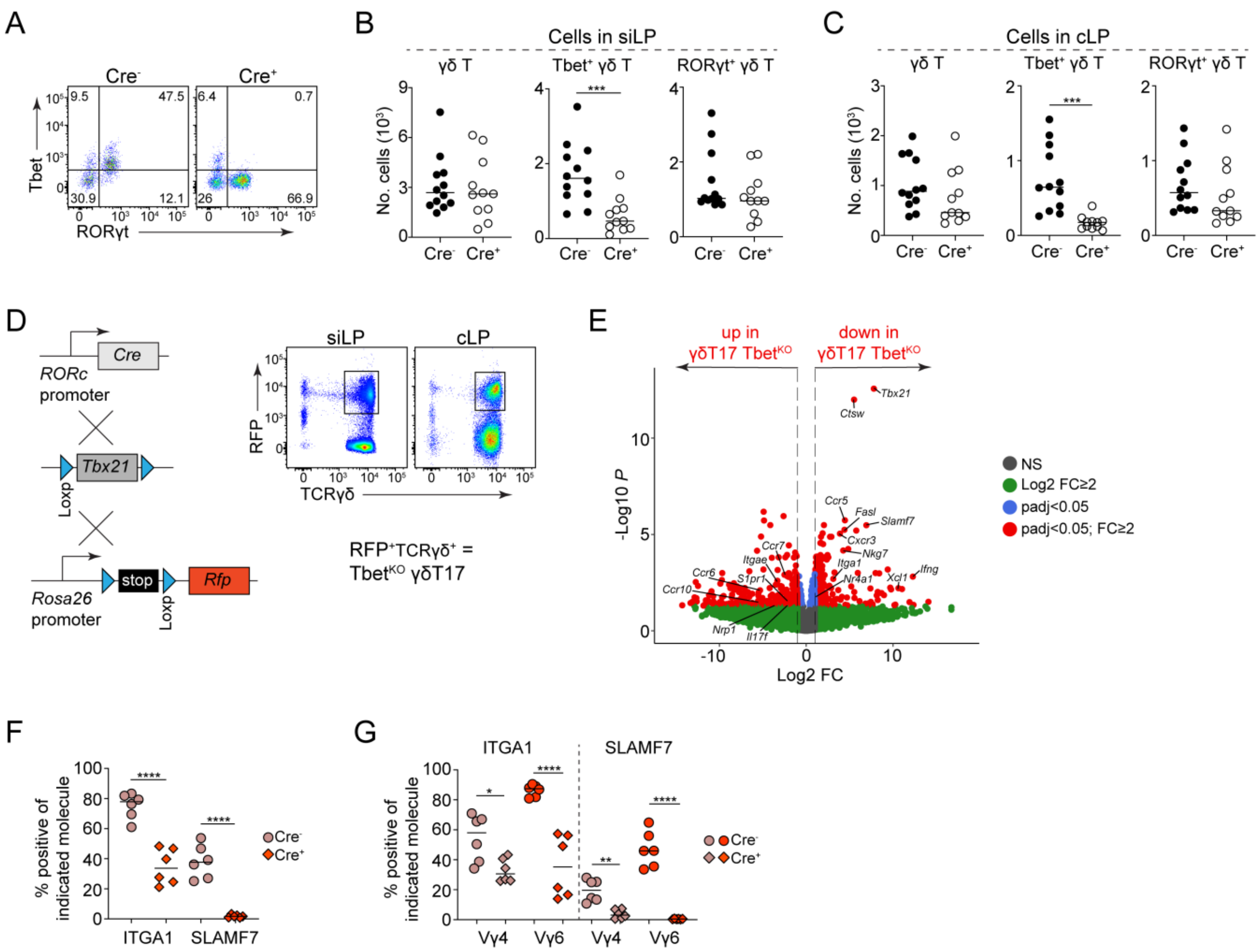


**Figure S4. Role of Tbet in intestinal γδ T cells.**

(**A**) Representative flow cytometric analysis of the expression of RORγt and Tbet in Tbet$^{F/F}$ (Cre$^{-}$) and RORγt$^{CRE}$-Tbet$^{F/F}$ (Cre$^{+}$) mice. (**B-C**) Numbers of total, Tbet$^{+}$ and RORγt$^{+}$ γδ T cells in siLP (**B**) and cLP (**C**) from Cre$^{-}$ and Cre$^{+}$ mice; data from 4 experiments with 11 or 12 mice. (**D**) Schematic representation of the genetic design used to generate Tbet-deficient (Tbet$^{KO}$) γδ T cells that were RFP$^{+}$ and representative flow cytometry plots showing the populations that were sorted from the LP. (**E**) Volcano plot indicating DEGs between Tbet-deficient (Tbet$^{KO}$) and WT γδT17 cells from cLP; each dot represents a gene; NS: not significant; selected genes highly expressed in either Tbet$^{KO}$ or WT are labeled on the plot; data from 3 biological repeats, each consisting of a pool of 4 mice. (**F-G**) Quantification of expression of the indicated markers in siLP RORγt$^{+}$ γδ T cells from Cre$^{-}$ and Cre$^{+}$ mice (**F**) and quantification of expression of the indicated markers in siLP Vγ4$^{+}$ and Vγ6$^{+}$ RORγt$^{+}$ γδ T cells from Cre$^{-}$ and Cre$^{+}$ mice (**G**); data from 2 experiments with 6 mice. In all graphs each symbol represents a mouse and line the median. P values were calculated using the

Mann-Whitney test in (**B-C**), a quasi-likelihood ratio test with the R package edgeR in (**E**), one-way ANOVA with Tukey's multiple comparisons correction in (**F**) and two-way ANOVA with Fisher's LSD multiple-comparisons test in (**G**); *, $P < 0.05$; **, $P < 0.01$; ***, $P < 0.001$; ****, $P < 0.0001$.

# Figure S5

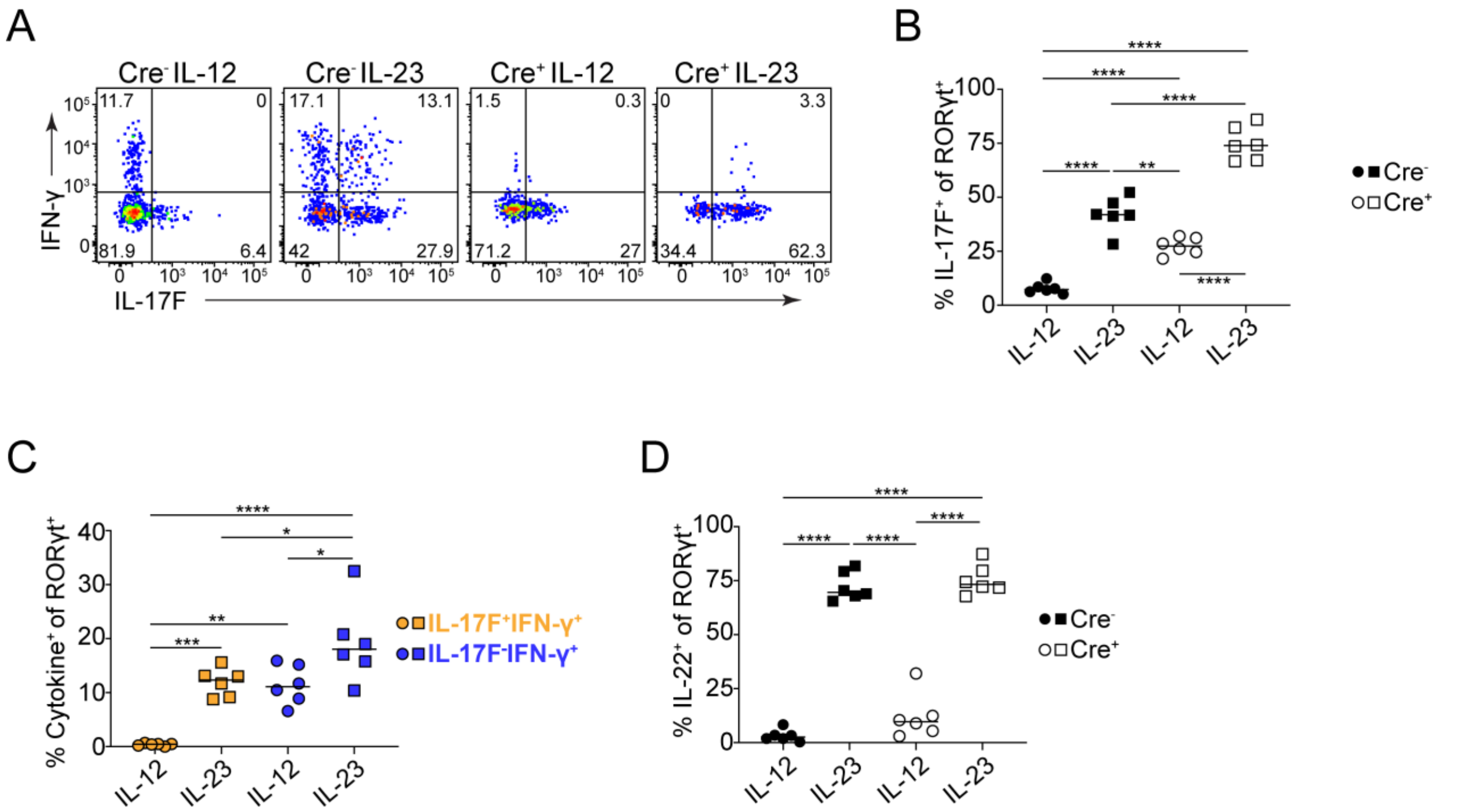


**Figure S5. Tbet differentially regulates IFN-γ and IL-17F but not IL-22 production.**

(**A**) Representative flow cytometric analysis of IFN-γ and IL-17A production in siLP $RORγt^+$ γδ T cells from $Tbet^{F/F}$ ($Cre^-$) and $RORγt^{CRE}$-$Tbet^{F/F}$ ($Cre^+$) mice after stimulation with IL-23 or IL-12. (**B**) Quantification of IL-17F levels in $Cre^-$ and $Cre^+$ siLP $RORγt^+$ γδ T cells after stimulation with IL-23 or IL-12. (**C**) Frequencies of IL-$17F^+$IFN-$γ^+$ (yellow) and IL-$17F^-$IFN-$γ^+$ (blue) $RORγt^+$ γδ T cells after stimulation with IL-23 or IL-12. (**D**) Quantification of IL-22 levels in $Cre^-$ and $Cre^+$ siLP $RORγt^+$ γδ T cells after stimulation with IL-23 or IL-12. Data from 2 experiments with 6 mice. In all graphs each symbol represents a mouse and line the median. P values were calculated using one-way ANOVA with Tukey's multiple comparisons correction; *, $P < 0.05$; **, $P < 0.01$; ***, $P < 0.001$; ****, $P < 0.0001$.

Figure S6

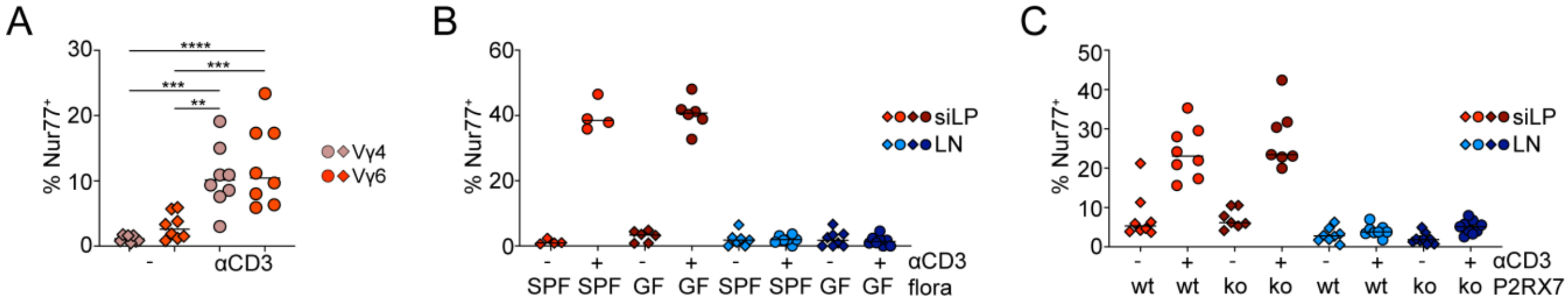


**Figure S6. Nur77 expression in intestinal γδT17 cells and role of P2RX7 and the microbiota.** (**A**) Quantification of Nur77 expression in Vγ4⁺ and Vγ6⁺ γδT17 cells after a 3-hour culture with or without αCD3; data from 2 experiments with 8 mice. (**B-C**) Quantification of Nur77 expression after a 3-hour culture with or without αCD3 in siLP and LN γδT17 cells from SPF and GF mice (**B**) or from WT and P2RX7$^{-/-}$ mice (**C**); data from a pool of 2 (**B**) or 3 (**C**) experiments including 4-8 mice (**B**) and 7-9 mice (**C**). In all graphs each symbol represents a mouse and line the median. P values were calculated using one-way ANOVA with Tukey's multiple comparisons correction; **, $P < 0.01$; ***, $P < 0.001$; ****, $P < 0.0001$.

Figure S7

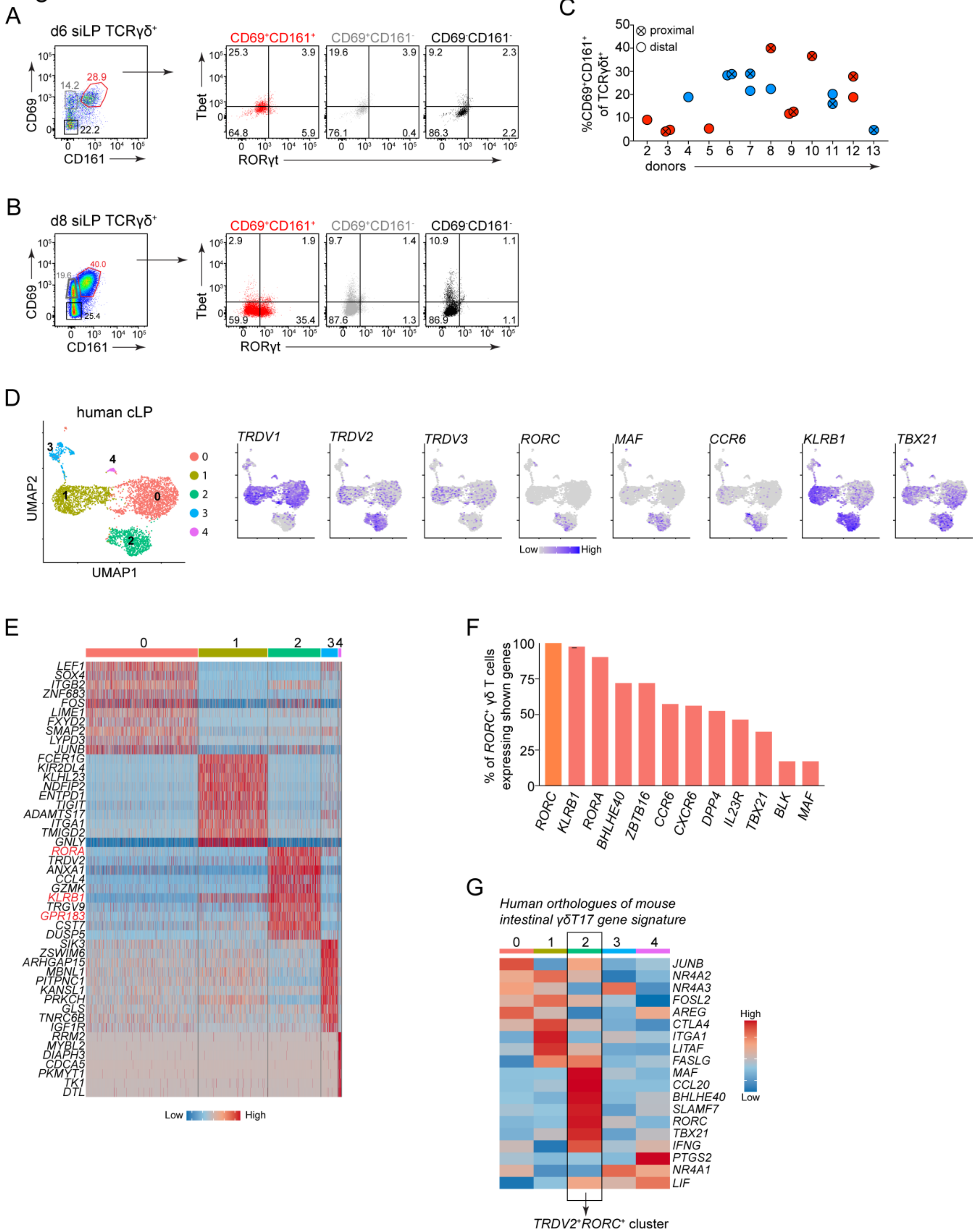


**Figure S7. RORγt-expressing type 3 γδ T cells in the human neonatal colon.**

(**A-B**) Representative flow cytometric analysis of human neonatal siLP from donor 6 (d6) (**A**) and donor 8 (d8) (**B**) indicating the presence of CD69⁺CD161⁺, CD69⁺CD161⁻ and CD69⁻CD161⁻ γδ T

cells and their expression of RORγt and Tbet. (**C**) Quantification of $CD69^+CD161^+$ cells within total γδ T cells across all donors; red = detectable $ROR\gamma t^+$ cells, blue = not detectable $ROR\gamma t^+$ cells. (**D**) UMAP showing how γδ T cell populations cluster in the human neonatal cLP and feature plots showing log-normalized expression levels of *TRDV1*, *TRDV2*, *TRDV3, RORC, MAF, CCR6, KLRB1*, and *TBX21* among these clusters. (**E**) Heatmap showing the top 10 DEGs for each γδ T cell cluster in the human neonatal cLP; all shown genes were significantly expressed for each cluster ($P < 0.05$; calculated using the Wilcoxon Rank Sum test) with a cut-off expression of $Log2FC \geq 1$; gene names in red indicate relevance to type 3 immunity. (**F**) Graphical representation of the frequency of *RORC*$^+$ cells expressing the indicated genes in human neonatal cLP; bars indicate the percentage of *RORC*-expressing cells with measurable (>0 counts) expression of each gene; genes are ordered by decreasing prevalence. (**G**) Heatmap showing, for each cluster, the expression level of the human orthologue genes of the mouse intestinal γδT17 signature that we defined in **Fig. 3D**.

# Figure S8

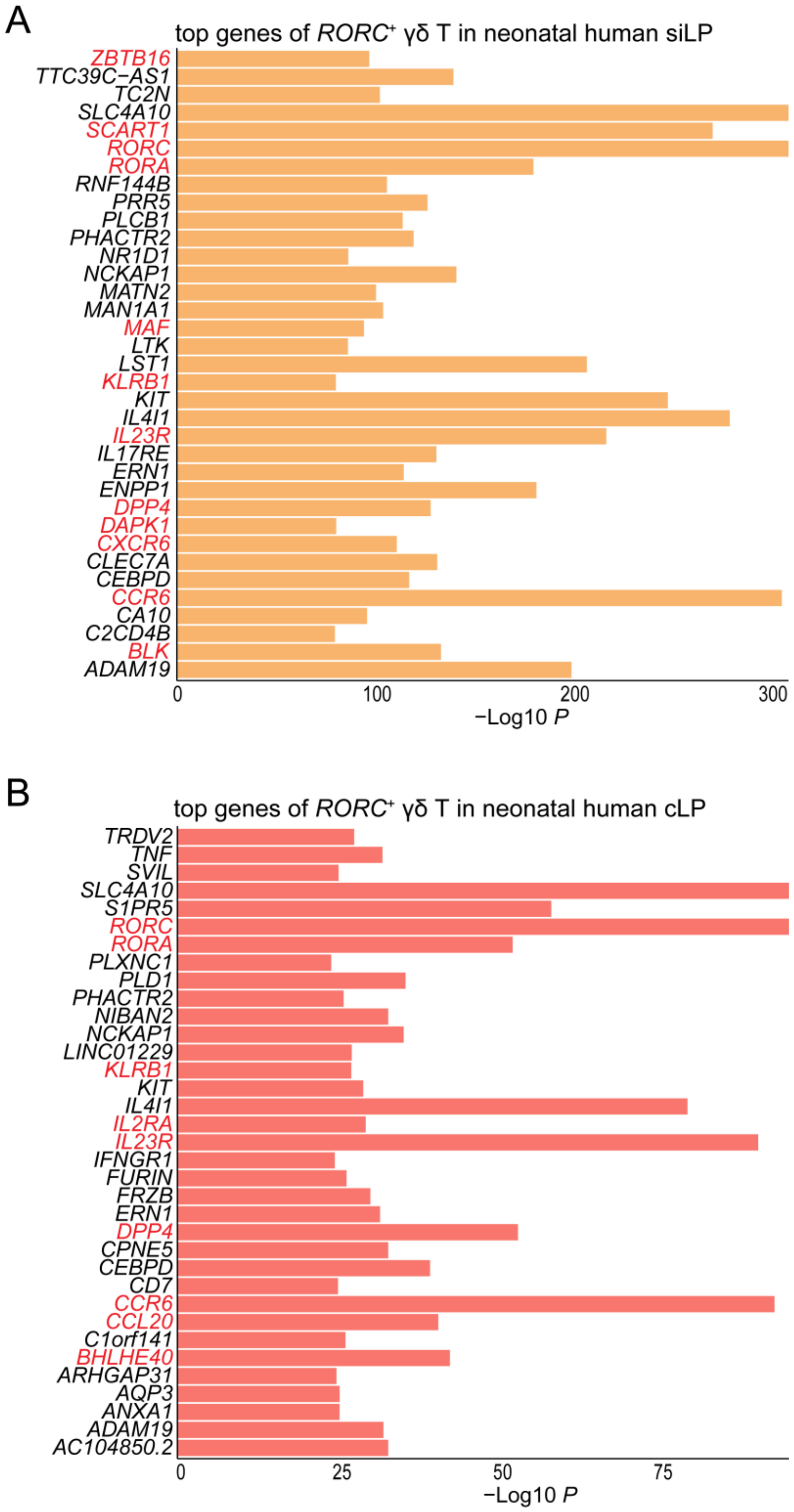


**Figure S8. Top genes expressed by neonatal *RORC*⁺ intestinal γδ T cells in siLP and cLP.**

(**A-B**) Graphical representation of the top 35 genes co-expressed with *RORC* in neonatal human siLP (**A**) or cLP (**B**) γδ T cells. Gene names in red indicate relevance to type 3 immunity. P values were calculated using Pearson correlation and multiple testing correction was applied using the Bonferroni method.

## Figure S9

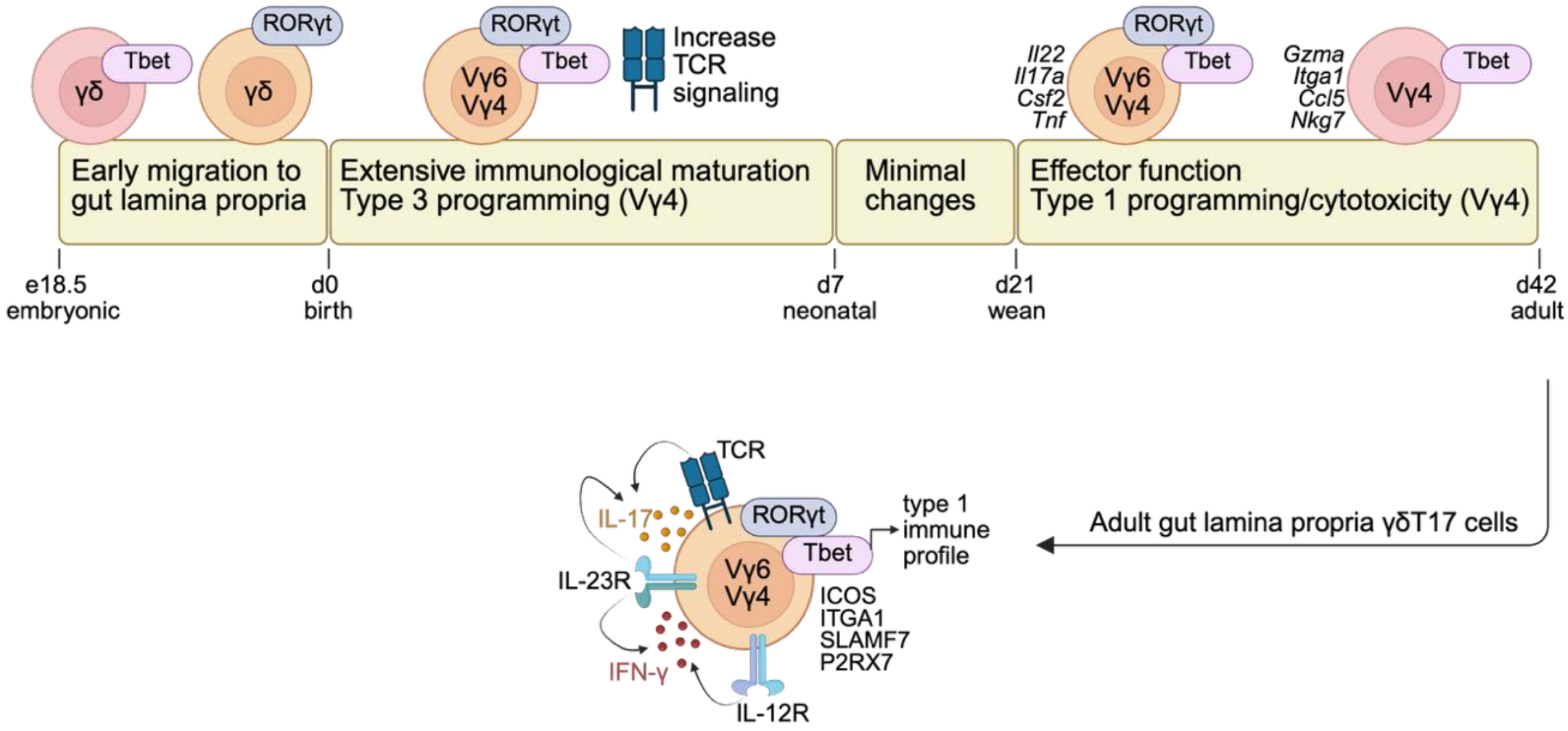


**Figure S9. Schematic overview of the proposed temporal differentiation program of intestinal lamina propria γδ T cells in mice.** γδ T cells develop in the thymus and migrate to the gut during embryonic and neonatal life. Subsequently, γδT17 cells upregulate Tbet and increase their TCR signaling capacity. During the neonatal period, both Vγ6 and Vγ4 subsets undergo an extensive immunological maturation, with Vγ4 cells displaying a marked type 3 programming. Following weaning, both subsets acquire further effector functions, and Vγ4 cells upregulate type 1 and cytotoxic genes. In adulthood, intestinal γδT17 cells exhibit type 1 properties in a Tbet-dependent manner and show a distinct functional and phenotypic profile. Tbet promotes and maintains type 1 programming while restraining IL-17 production. Functionally, γδT17 cells acquire the capacity to produce IFN-γ in response to IL-12 and IL-23, which is Tbet-dependent. IL-23 is particularly capable of promoting the co-production of IFN-γ and IL-17. Created in BioRender. Bekiaris, V. (2026) https://BioRender.com/qg7awwz.

## Captions for Supplementary Tables

**Table S1. Clustering of gut γδT cells identified by single-cell RNA sequencing after integration of all time points.** Single-cell RNA sequencing data from intestinal γδT cells were integrated across all analyzed time points and subjected to unsupervised clustering. The table shows the identified clusters, with one worksheet per cluster containing the corresponding list of genes.

**Table S2. Differentially expressed genes in Vγ6 and Vγ4 cells identified by single-cell RNA sequencing.** The table presents comparisons of differentially expressed genes (DEGs) in Vγ6 and Vγ4 cells between timepoints. Separate worksheets are provided for each comparison: day (d)0 V d7; d7 V d21; d21 V d42; d7 V d42. Only genes with statistically significant differential expression ($P < 0.05$) and a log2 fold change (Log2FC) ≥ 1 are included.

**Table S3. Differentially expressed genes in mouse tissues identified by bulk RNA sequencing.** The table presents comparisons of DEGs between timepoints, mouse tissues and genotypes. Separate worksheets are provided for each comparison: gut γδT17 d0 V d7; γδT17 LN V siLP; γδT17 LN V cLP; γδT17 siLP V cLP; siLP $Tbet^{KO}$ V WT; cLP $Tbet^{KO}$ V WT. Only genes with statistically significant differential expression ($P < 0.05$) and Log2FC ≥ 1 are included.

**Table S4. Single-cell RNA sequencing analysis of human samples.** The table presents the clustering of γδT cells in the human neonatal small intestine lamina propria (siLP) and colon lamina propria (cLP), as well as the top DEGs among clusters within siLP and cLP. Only genes with statistically significant differential expression ($P < 0.05$) and Log2FC ≥ 1 are included.

**Data S1**

The file “Data S1.xlsx” contains the tabulated data underlying all main and supplementary figures. Each figure has its own worksheet, appropriately named and arranged in order of appearance.
